# Exploring a novel β-1,3-glucanosyltransglycosylase, *Ml*GH17B, from a marine *Muricauda lutaonensis* strain for modification of laminari-oligosaccharides

**DOI:** 10.1093/glycob/cwae007

**Published:** 2024-01-25

**Authors:** Leila Allahgholi, Maik G N Derks, Justyna M Dobruchowska, Andrius Jasilionis, Antoine Moenaert, Léonie Jouy, Kazi Zubaida Gulshan Ara, Javier A Linares-Pastén, Ólafur H Friðjónsson, Guðmundur Óli Hreggviðsson, Eva Nordberg Karlsson

**Affiliations:** Division of Biotechnology, Department of Chemistry, Lund University, PO Box 124, Lund SE-221 00, Sweden; Division of Biotechnology, Department of Chemistry, Lund University, PO Box 124, Lund SE-221 00, Sweden; Department of Chemical Biology and Drug Discovery, Utrecht Institute for Pharmaceutical Sciences, Bijvoet Center for Biomolecular Research, Utrecht University, Universiteitsweg 99, Utrecht 3584 CG, The Netherlands; Division of Biotechnology, Department of Chemistry, Lund University, PO Box 124, Lund SE-221 00, Sweden; Department of Biotechnology and Biomedicine, Matís ohf, Vínlandsleið 12, Reykjavík IS-113, Iceland; Faculty of Life and Environmental Sciences, University of Iceland, Sturlugata 7, Reykjavík IS-102, Iceland; Department of Biotechnology and Biomedicine, Matís ohf, Vínlandsleið 12, Reykjavík IS-113, Iceland; Division of Biotechnology, Department of Chemistry, Lund University, PO Box 124, Lund SE-221 00, Sweden; Division of Biotechnology, Department of Chemistry, Lund University, PO Box 124, Lund SE-221 00, Sweden; Department of Biotechnology and Biomedicine, Matís ohf, Vínlandsleið 12, Reykjavík IS-113, Iceland; Department of Biotechnology and Biomedicine, Matís ohf, Vínlandsleið 12, Reykjavík IS-113, Iceland; Faculty of Life and Environmental Sciences, University of Iceland, Sturlugata 7, Reykjavík IS-102, Iceland; Division of Biotechnology, Department of Chemistry, Lund University, PO Box 124, Lund SE-221 00, Sweden

**Keywords:** β-1, 3-glucanosyltransglycosylase, carbohydrate biosynthesis, laminari-oligosaccharides, Muricauda lutaonensis, three-dimensional modeling

## Abstract

The marine environment, contains plentiful renewable resources, e.g. macroalgae with unique polysaccharides, motivating search for enzymes from marine microorganisms to explore conversion possibilities of the polysaccharides. In this study, the first GH17 glucanosyltransglycosylase, *Ml*GH17B, from a marine bacterium (*Muricauda lutaonensis*), was characterized. The enzyme was moderately thermostable with T_m_ at 64.4 °C and 73.2 °C, but an activity optimum at 20 °C, indicating temperature sensitive active site interactions. *Ml*GH17B uses β-1,3 laminari-oligosaccharides with a degree of polymerization (DP) of 4 or higher as donors. Two glucose moieties (bound in the aglycone +1 and +2 subsites) are cleaved off from the reducing end of the donor while the remaining part (bound in the glycone subsites) is transferred to an incoming β-1,3 glucan acceptor, making a β-1,6-linkage, thereby synthesizing branched or kinked oligosaccharides. Synthesized oligosaccharides up to DP26 were detected by mass spectrometry analysis, showing that repeated transfer reactions occurred, resulting in several β-1,6-linked branches. The modeled structure revealed an active site comprising five subsites: three glycone (−3, −2 and −1) and two aglycone (+1 and +2) subsites, with significant conservation of substrate interactions compared to the only crystallized 1,3-β-glucanosyltransferase from GH17 (*Rm*Bgt17A from the compost thriving fungus *Rhizomucor miehei*), suggesting a common catalytic mechanism, despite different phylogenetic origin, growth environment, and natural substrate. Both enzymes lacked the subdomain extending the aglycone subsites, found in GH17 endo-β-glucanases from plants, but this extension was also missing in bacterial endoglucanases (modeled here), showing that this feature does not distinguish transglycosylation from hydrolysis, but may rather relate to phylogeny.

## Introduction

Macroalgae from the marine environment include fast growing species with potential as resources for next generation biorefineries. Brown macroalgae are of interest, due to high bulk biomass production and high carbohydrate content. One of the main types of polysaccharides in brown macroalgae is laminarin, which is a storage β-glucan (Hakim and Patel 2020; Konstantin et al. 2023). Glucans are polysaccharides derived from d-glucose, linked by glycosidic bonds. Laminarin is a low molecular weight β-1,3-d-glucan polymer with sporadic β-1,6-linkages and a degree of polymerization (DP) of 20–35 glucose units (Becker et al. 2020). The reducing ends are capped with either a mannitol or a glucose moiety, and the extent of branching is important for the solubility of the polymer. Other types of β-1,3-linked glucans can be found in the cell walls of terrestrial plants, bacteria and fungi, including callose (from plants), and curdlan (from the bacterial family *rhizobiaceae*), which are not reported to contain 1,6-linkages, while β-1,6-linkages can be found in β-1,3-linked glucans from baker’s yeast (Sajna et al. 2015). Laminarin is mainly found in the brown seaweed genera *Laminaria* and *Saccharina*, but also to a lesser extent in other species in the genera *Ascophyllum* and *Undaria* (Kadam et al. 2015). Currently there is an increased interest in polymers from seaweeds, as it is a biomass that does not compete with arable land. In relation to this, the interest in laminarin, and laminarin-derived oligosaccharides, has also increased as these compounds can be expected to elicit the same health-promoting responses as reported for other β-glucans (Jönsson et al. 2020).

Laminarin can be selectively converted to oligosaccharides by controlled enzymatic action. Endo-acting enzymes are in this aspect of interest, as they can be utilized in production of value-added laminari-oligosaccharides (Chesters and Bull 1963; Dobruchowska et al. 2016). Candidates catalyzing endohydrolysis of β-1,3 glucans (EC 3.2.1.39) have, based on sequence similarities, been classified under ten different glycoside hydrolase families (GHs) according to the CAZy database (cazy.org, visited 2023-10-11) (Drula et al. 2022), but only a few families have consistent laminarinase (or β-1,3 glucanase) activity. In GH5, GH16, and GH55, only few candidates display activity on laminarin, interspersed with enzymes holding other types of glucanase activities. Additionally, 18 characterized enzymes are available in GH64, GH128, GH152, GH157, and GH158 families. This leaves GH17 (53 characterized enzymes) and GH81 (15 characterized enzymes) as the two families with most consistent activity on laminarin or other glucans including β-1,3-linkages.

The GH17 family mainly consists of hydrolyzing enzymes with retaining catalytic mechanism, including endo β-1,3 glucanases, exo β-1,3 glucanases, and mixed-linkage endo (β-1,3 and β-1,4) glucanases (Thomas et al. 2000). Members of the GH17 family have a TIM-barrel ((β/α)_8_) fold, and are classified under GH clan A (cazy.org, visited 2023-10-11). Two Glu residues serve as the catalytic nucleophile and acid/base (Drula et al. 2022).

Despite the large number of characterized enzymes in the GH17 family, the large majority are of eukaryotic origin and to date, only eight candidates of bacterial origin are listed in the CAZy database (visited 2023-10-11), which all originate from terrestrial habitats. Interestingly, among these eight enzymes, two distinct activity groups can be distinguished: glucan endo-1,3-β-glucosidases (EC 3.2.1.39) and transglycosylases (EC 2.4.1.-). The transglycosylases are few, and to date the identified bacterial GH17-transglycosylases all originate from proteobacteria from soil habitats (Hreggvidsson et al. 2011; Linares-Pastén et al. 2021). In addition to this, two fungal transglycosylases can be found in GH17, originating from *Aspergillus fumigatus* (Okada et al. 1998) and *Rhizomucor miehei* (Qin et al. 2015). None of the previously identified transglycosylases, originate from marine environments.

**Fig. 1:**
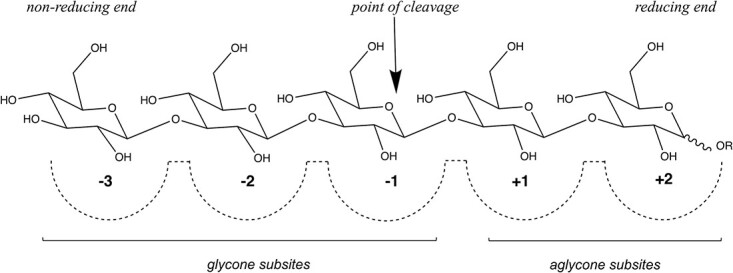
Nomenclature for sugar binding subsites in *Ml*GH17B. The non-reducing end of the donor substrate is bound to subsite −3 while the reducing end is bound to subsite +2. After cleavage and release of the Glc_2_ product, the donor substrate remains in the glycone subsites (−3,−2, and −1subsites) while the acceptor substrate is bound to the aglycone subsites (+1 and +2) in an alternative position for the transglycosylation reaction to happen.

The five listed GH17 transglycosylases cleave the donor substrate and transfer the remaining part to a new substrate molecule, but the type and position of the new linkage is dependent on the enzyme, and include: β-1,3-elongation (Hartland et al. 1996; Hreggvidsson et al. 2011), β-1,4-elongation (Hreggvidsson et al. 2011), and β-1,6-elongation or branching (Gastebois et al. 2010; Hreggvidsson et al. 2011; Dobruchowska et al. 2016; Aimanianda et al. 2017). Elongation implies transfer of the bound glycone (the donor) to the non-reducing end of an incoming substrate molecule bound to the aglycone subsites (the acceptor) (Fig. 1), while branching implies transfer to a sugar moiety, inside the incoming aglycone chain. Due to the limited number of characterized transglycosylating enzymes, and the variations in product profiles of the so far characterized enzymes, more information on GH17 transglycosylases is needed, especially considering the lack of characterized marine candidates.

In this study, a novel GH17 glucanosyltransglycosylase, from *Muricauda lutaonensis*, a moderately thermophilic marine bacterium, classified as a member of the family *Flavobacteriaceae* (Arun et al. 2009a), known to contain many efficient biomass degraders, was subjected to thorough characterization. Selection of a moderately thermophilic species was made as enzymes from these sources often display stability for application purposes. The product profile of the recombinant enzyme was investigated in the presence of laminarin and laminari-oligosaccharides (Glc_2-6_) by employing high-performance anion exchange chromatography with pulsed amperometric detection (HPAEC-PAD) and thin layer chromatography (TLC) methods. The enzyme’s ability in branch formation and production of long chain oligosaccharides was investigated by matrix-assisted laser desorption ionization time-of-flight (MALDI-TOF) as well as one-dimensional and two-dimensional nuclear magnetic resonance (NMR) spectroscopy. Furthermore, bioinformatic analysis, including modeling of the three-dimensional structure, was performed to gain more insight into the structure and catalytic cleft of the enzyme, supporting it as the first GH17-transglycosylase isolated from a marine environment.

## Results

### Sequence analysis of the 16S rRNA gene, and the identification of the gene encoding *Ml*GH17B in strain ISCAR-4703

In order to find marine bacteria with a potential to produce enzymes with sufficient stability, sampling for candidate species was made at the coastal, intertidal, marine geothermal Yngingarlindir site, rich in seaweeds, located off the coast of the Reykjanes peninsula (Iceland). This was followed by isolation of aerobic species using marine agar, as detailed in materials and methods. Genomic DNA was then prepared from the isolate (termed ISCAR-4703) followed by sequencing and assembly into a draft genome. The amplified and partially sequenced 16S rRNA gene demonstrated >99% identity to the 16S rRNA gene (GenBank EU564844.1) of *M. lutaonensis* which is a moderately thermophilic member of the family *Flavobacteriaceae* (Arun et al. 2009b) and allowed classification of the strain as *M. lutaonensis* ISCAR-4703*.* The finding that the isolated bacterium was a member of the genus *Muricauda* was interesting as members from this genus are relatively understudied,

To find GH17 candidates, the genome was subsequently searched for homologs to identified transglycosylases, allowing identification of the gene encoding *Ml*GH17B. Sequence similarity search by blastp, with *Ml*GH17B as query sequence against the non-redundant protein sequence database demonstrated that the encoded amino acid sequence of *Ml*GH17B (GenBank WIW39500.1) was identical over the complete 295 residue sequence to a gene annotated as a putative glycosyl hydrolase (GenBank MBC31414.1) in the draft genome (GenBank PARJ01000001.1) of *Muricauda* sp. SP11 identified from a marine metagenome from the *Tara* global ocean expedition (Tully et al. 2018). The enzyme lacks signal peptide, and suggests that it is cytoplasmic with a potential role in the cell’s energy storage.

The blastp-search also revealed a large number of deposited genes that encoded homologous putative enzymes from the GH17 family. The deduced amino acid sequences of the 30 best matches, displayed sequence identities to *Ml*GH17B ranging from 74.6% to 100%, over 100% query coverage. Most of these homologous genes were originating from various *Muricauda* species, demonstrating that this type of single module enzyme appears common among related bacteria in the marine environment. None of these genes has however, been cloned or its gene product characterized.

Sequence similarities to characterized enzymes were significantly lower. Candidates found by blastp in the Swiss-Prot database, for example resulted in a best match with a glucan-1,3-glucosidase (Swiss-Prot P15703.1) from *Saccharomyces cerevisiae*, with only 27% identity over 78% query coverage. Blastp search in the Protein Data Bank (PDB), demonstrated that the most similar structure determined candidate was the fungal GH17 enzyme designated as a 1,3-β-glucanosyltransferase *Rm*Bgt17A (PDB 4WTP) from *R. miehei* with 33.8% identity over 77% query coverage.

**Fig. 2:**
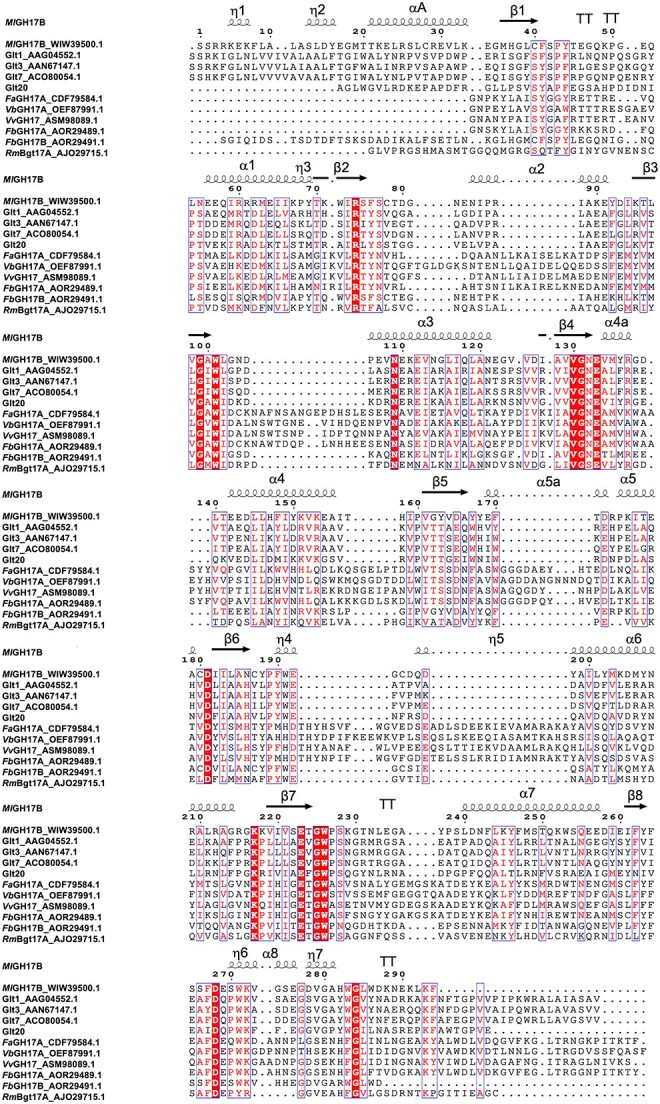
Multiple sequence alignment of the catalytic modules of all characterized GH17 family bacterial transglycosylases and hydrolases as well as fungal *Rm*Bgt17A from *Rhizomucor miehei*. *Ml*GH17B, β-1,3-glucanosyltransglycosylase from *M. lutaonensis* ISCAR-4703 (GenBank WIW39500.1); Glt1 (GenBank AAG04552.1), β-1-3-glucan synthase from *Pseudomonas aeruginosa*; Glt3 (GenBank AAN67147.1), glucanosyltransglucosidase from *pseudomonas putida*; Glt7 (GenBank ACO80054.1), glucanosyltransglucosidase from *Azotobacter vinelandii*; Glt20, transglycosylase from *Bradyrhizobium diazoefficiens*; *Fa*GH17A (GenBank CDF79584.1), laminarinase from *Formosa agariphila*; *Vb*GH17A (GenBank OEF87991.1), laminarinase from *vibrio breoganii*; *Vv*GH17 (GenBank ASM98089.1), endo-β-1,3-glucanase from *Vibrio vulnificus*; *Fb*GH17A (GenBank AOR29489.1), laminarinase from *Formosa* sp. Hel1_33_131; *Fb*GH17B (GenBank AOR29491.1), laminarinase from *Formosa* sp. Hel1_33_131; *Rm*Bgt17A (GenBank AJO29715.1), β-1,3-glucanosyltransferase from *Rhizomucor miehei*. The predicted structure of *Ml*GH17B using AlphaFold2 is outlined on top of the alignment indicating β sheets,α helices, 310-helix marked as η, and strict β-turns marked as TT. αA is the N-terminal α helices located before β1 sheet. Conserved residues are presented on red background columns, and identical residues are shown in red.

Surprisingly, the blastp searches did not result in significant matches with the bacterial enzymes, listed as characterized in the CAZy database (cazy.org, 2023-10-11) (Drula et al. 2022), from which the sequences of the catalytic modules were originally used to find the gene in the genome sequence. The lack of hits is judged to be due to that these enzymes are deposited in their original multi-modular form, resulting in low query coverage. To further display similarities and differences, an alignment of the catalytic modules of the characterized bacterial enzymes, in the sequence-format used for recombinant production (Linares-Pastén et al. 2021), was generated. The alignment also included the fungal transglycosylase *Rm*Bgt17A, and the catalytic module of the bacterial transglycosylase Glt20 (originating from the soil-bacterium *Bradyrhizobium diazoefficiens*, despite not yet being indexed as characterized in the CAZy database, visited 2023-10-11) (Fig. 2). It is noteworthy to mention that both *Rm*Bgt17A and Glt20 show β-1,6-elongation or branching, in line with the activity found for *Ml*GH17B (see below).

*Ml*GH17B is shown to be a single-domain enzyme, consisting only of a catalytic module, whereas the characterized proteins from Proteobacteria (termed Glt1, Glt3, Glt7) (Hreggvidsson et al. 2011) have an N-terminal domain belonging to GH17 and a C-terminal domain belonging to the Leloir glycosyltransferase family GT2 (Hreggvidsson et al. 2011). The alignment demonstrated that sequence identities to *Ml*GH17B were rather low, and in the same range for the catalytic modules of transglycosylases from proteobacteria and for the single module fungal transglycosylase *Rm*Bgt17A (Table 1), although the proteobacterial sequences shared higher sequence similarities with each other, than with either *Ml*GH17B or *Rm*Bgt17A (Table 1), indicating some differences in conserved residues. Moreover, the sequence identity (16%–22%) was even lower between *Ml*GH17B and bacterial laminarinases/endo-β-1,3 glucanases (Badur et al. 2020; Kumagai et al. 2022).

**Table 1:** Percent identity matrix of all characterized bacterial GH17 family both transglycosylases and hydrolases as well as fungal *Rm*Bgt17A from *Rhizomucor miehei*. *Ml*GH17B, β-1,3-glucanosyltransglycosylase from *M. lutaonensis* ISCAR-4703 (GenBank WIW39500.1); Glt1 (GenBank AAG04552.1), β-1-3-glucan synthase from *Pseudomonas aeruginosa*; Glt3 (GenBank AAN67147.1), glucanosyltransglucosidase from *pseudomonas putida*; Glt7 (GenBank ACO80054.1), glucanosyltransglucosidase from *Azotobacter vinelandii*; Glt20, transglycosylase from *Bradyrhizobium diazoefficiens*; *Fa*GH17A (GenBank CDF79584.1), laminarinase from *Formosa agariphila*; *Vb*GH17A (GenBank OEF87991.1), laminarinase from *vibrio breoganii*; *Vv*GH17 (GenBank ASM98089.1), endo-β-1,3-glucanase from *Vibrio vulnificus*; *Fb*GH17A (GenBank AOR29489.1), laminarinase from *Formosa* sp. Hel1_33_131; *Fb*GH17B (GenBank AOR29491.1), laminarinase from *Formosa* sp. Hel1_33_131; *Rm*Bgt17A (GenBank AJO29715.1).

	***Ml*GH17B_WIW39500.1**	**Glt1_AAG04552.1**	**Glt3_AAN67147.1**	**Glt7_ACO80054.1**	**Glt20**	***Fa*GH17A_CDF79584.1**	***Vb*GH17A_OEF87991.1**	***Vv*GH17A_ASM98089.1**	***Fb*GH17A_AOR29489.1**	***Fb*GH17B_AOR29491.1**	***Rm*Bgt17A_AJO29715.1**
***Ml*GH17B_WIW39500.1** β-glucosyltransferase	100	28.57	26.53	27.89	30.69	21.92	16.35	21.15	21.92	53.17	28.73
**Glt1_AAG04552.1** Cyclic β-1-3-glucan synthase	28.57	100	73.21	70.40	41.46	21.43	20.00	24.47	23.57	27.56	24.29
**Glt3_AAN67147.1** Glucanosyltransglucosidase	26.53	73.21	100	80.06	43.55	25.00	21.05	24.47	23.57	26.15	22.50
**Glt7_ACO80054.1** Glucanosyltransglucosidase	27.89	70.40	80.06	100	45.30	24.64	22.81	25.18	24.29	29.68	23.93
**Glt20** β-glucosyltransferase	30.69	41.46	43.55	45.30	100	26.69	21.03	22.01	23.68	28.15	26.57
***Fa*GH17A_CDF79584.1** Laminarinase	21.92	21.43	25.00	24.64	26.69	100	52.91	53.63	67.96	19.76	20.60
***Vb*GH17A_OEF87991.1 L**aminarinase	16.35	20.00	21.05	22.81	21.03	52.91	100	57.85	51.25	18.75	17.28
***Vv*GH17A_ASM98089.1** Endo-β-1,3-glucanase	21.15	24.47	24.47	25.18	22.01	53.63	57.85	100	51.68	20.16	21.19
***Fb*GH17A_AOR29489.1** Laminarinase	21.92	23.57	23.57	24.29	23.68	67.96	51.25	51.68	100	20.95	18.73
***Fb*GH17B_AOR29491.1** Laminarinase	53.17	27.56	26.15	29.68	28.15	19.76	18.75	20.16	20.95	100	28.35
***Rm*Bgt17A_AJO29715.1** β-1,3-glucanosyltransferase	28.73	24.29	22.50	23.93	26.57	20.60	17.28	21.19	18.73	28.35	100

Interestingly, the highest sequence similarity, displaying 53% identity, was to a GH17 catalytic module of the two-domain enzyme (*F*bGH17B), from a marine bacterial *Formosa* sp. (GenBank AOR29491.1), that also included an MFS-family sugar-transporter (Unfried et al. 2018). The *Fb*GH17B is, however, despite its significant sequence similarity, proposed to be an exo-acting β-1,3-glucosidase, hydrolyzing debranched laminarin, producing glucose (Unfried et al. 2018).

### Production and purification of the recombinant *Ml*GH17B

The relatively low sequence homologies to characterized enzymes, the low number of characterized transglycosylases, and the wide product spectrum of the previously characterized transglycosylases in GH17, motivated exploring the structure and function of the novel identified *Ml*GH17B, which was subsequently produced using *Escherichia coli* as an expression host.

Production of a soluble entity was achieved by using an N-terminal MBP solubility tag, MBP-Smt3-*Ml*GH17B, that comprised the productivity of approximately 0.16 g/Lh of the soluble recombinant protein fraction produced by the host cells. Cleavage of MBP with Ulp1 protease (recognizing the Smt3 motif between MBP and the enzyme sequence) was possible, however *Ml*GH17B without MBP precipitated instantly and investigation of the recombinant enzyme properties necessitated use of the fusion-protein.

Purification of MBP-Smt3-*Ml*GH17B by affinity chromatography ensured a stable fusion-protein of high purity, as assessed by SDS-PAGE (Supplementary Fig. S1). The molecular weight of MBP-Smt3-*Ml*GH17B-6×His was estimated to 86 kDa, and comprised the MBP-Smt3 domain (54 kDa), the *Ml*GH17B (31 kDa), and the 6×His-tag (0.8 kDa) confirming the expected molecular weight for the complete target construct. Activity was only detected in fractions where the MBP-domain was not cleaved or only partly cleaved off from MBP-Smt3-*Ml*GH17B. Hence, the enzyme is not active after precipitation.

### Thermostability

The melting curve of MBP-Smt3-*Ml*GH17B exhibited two transition peaks, with a major peak at T_m1_ 64.4 ± 0.1 °C and a small peak at T_m2_ 73.2 ± 0.2 °C. The folded recombinant enzyme was stable up to T_onset_ 58.9 ± 0.1 °C, which is the temperature corresponding to the first transition start (Supplementary Fig. S2). The unfolding temperature of the purified MBP was found to be T_m1_ 64.4 ± 0.1 °C (Supplementary Fig. S2, red line); hence the MBP and *Ml*GH17B domains are judged to unfold at the same temperature, followed by a smaller unfolding event of some remaining structure at T_m2_ 73.2 ± 0.2 °C (Supplementary Fig. S2, blue line). The MBP melting point is in line with previous data, as estimated in the presence of 50 mM maltose, and it has been shown to vary between 59.3–72.1 °C as it is affected by buffer composition as well as buffer pH (Novokhatny and Ingham 1997).

### pH and temperature optimum

**Fig. 3:**
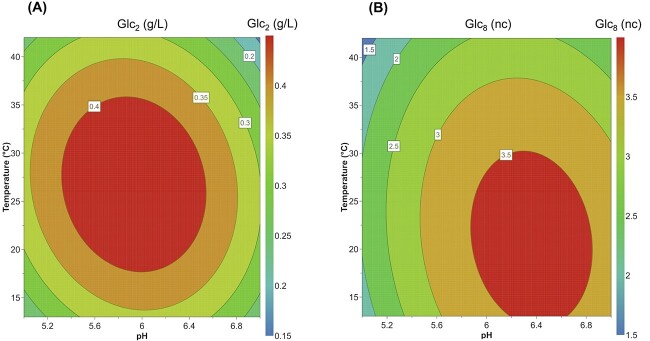
Response contour plot representing the effect of pH and temperature (°C) on product formation (Glc_2_ and Glc_8_) by *Ml*GH17B in the presence of Glc_5_ substrate. Glc_2_ and Glc_8_ represent laminaribiose (hydrolysis product) and laminarioctaose (transferase product), respectively, produced by *Ml*GH17B; nc (nanocoulombs) represents the HPAEC-PAD response.

Initial screening to find an optimal pH for activity was performed in the pH range 3.5–9 at room temperature using Glc_5_ as the substrate. This enabled estimation of the pH optimum of *Ml*GH17B to approximately pH 6.0. Further analysis was subsequently performed at a narrower pH range (pH 5, 5.5, 6, and 6.5) to confirm the pH optimum. Product formation was subsequently measured at a temperature range of 13–42 °C during 2 h reaction time, which resulted in estimated pH and temperature optima (Fig. 3) at pH 6.0 and 20 °C, respectively.

The activity of *Ml*GH17B was assayed with different oligo- and polysaccharides of varying length, branching, and glycosidic linkage types. The reaction mixtures were incubated at the optimum conditions for 24–72 h. Analysis by TLC and HPAEC-PAD, did not reveal any detectable activity on chitosan, xylan, lichenan, maltodextrin, amylose, malto-oligosaccharides, or gentiobiose. However, activity toward laminari-oligosaccharides (Glc_4-6_) and laminarin confirmed that the enzyme has high specificity toward glucan substrates with β-1,3-linkages.

**Fig. 4:**
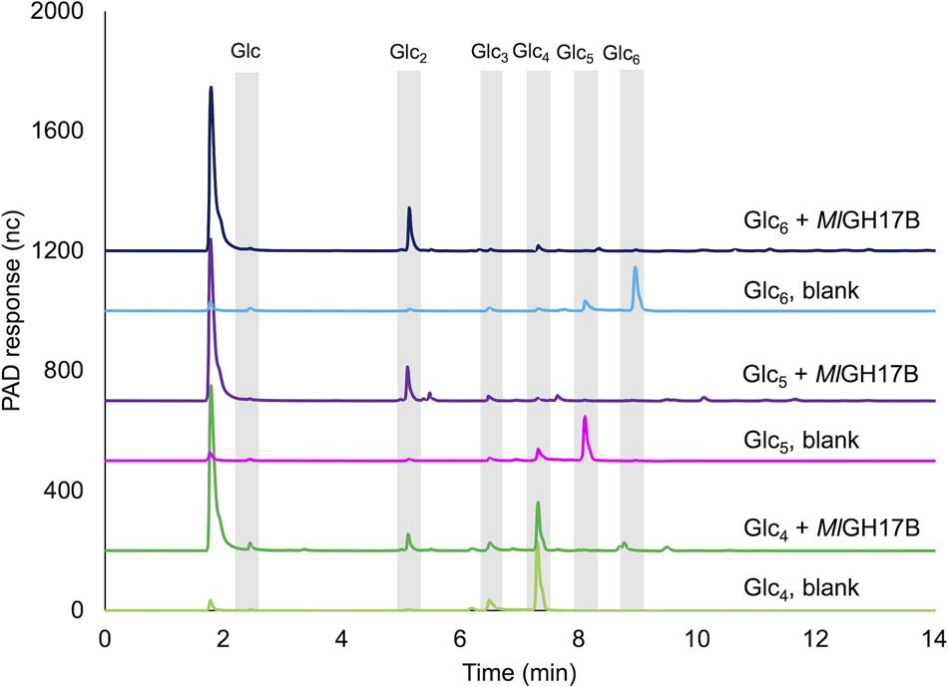
HPAEC-PAD analysis of *Ml*GH17B reaction products obtained with laminaritetraose (Glc_4_), laminaripentaose (Glc_5_), and laminarihexaose (Glc_6_), at pH 6.0 and 20 °C after 24 h of reaction incubation. The gray bars represent the elution time of glucose (Glc) and laminari-oligosaccharides standards (Glc_2–6_); nc (nanocoulombs) represents the HPAEC-PAD response.

The product profile obtained from HPAEC-PAD (Fig. 4) clearly illustrated that the enzyme was able to act on laminari-oligosaccharides with a DP of 4–6 (Glc_4_, Glc_5_, and Glc_6_). The activity on Glc_4_ was, however, very low compared to the activity on Glc_5_ and Glc_6_ which were completely utilized by the enzyme in the selected incubation period. The increase in intensity of the peak corresponding to the laminaribiose standard (Glc_2_) confirmed that the enzyme cleaved off two sugar units regardless of the size of the substrate. A Glc_2_ product was also observed after incubation with a laminarin polysaccharide, and the remaining part was transferred to another molecule of the substrate (or extended product), acting as acceptor substrate. However, detection of the transfer products was not possible using laminarin as substrate due to the complexity and size distribution of the polysaccharide substrate (Supplementary Fig. S3).

After a 24 h incubation of the enzyme with the substrates, a trace amount of the DP3 product, resulting from hydrolysis of the Glc_5_ substrate, and the DP4 product, resulting from hydrolysis of the Glc_6_ substrate were detected. However, the amount of these products were negligible, compared to the respective transferase products.

### MS analysis of transferase products of *Ml*GH17B

The reaction mixture of *Ml*GH17B with Glc_4_, Glc_5_, and Glc_6_, after 48 h incubation at the activity optimum condition, was analyzed by MS. MALDI-TOF mass spectra agreed well with the product profile of the enzyme, detected by HPAEC-PAD, demonstrating the release of Glc_2_ and the transfer of the remaining donor molecule to another incoming substrate molecule functioning as acceptor molecule.

**Fig. 5:**
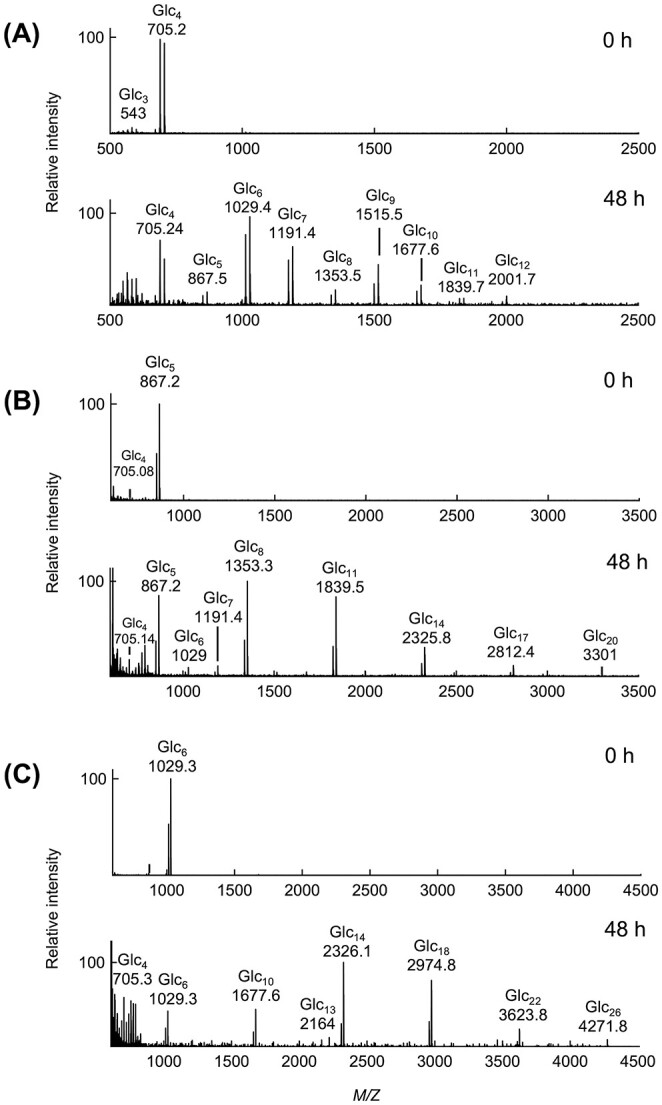
MALDI-TOF mass spectra corresponding the quasi-molecular ions [M + K]^+^ of the oligosaccharide mixtures, produced by *Ml*GH17B, at activity optimum conditions (pH 6.0 and 20 °C), using (A) Glc_4_, (B) Glc_5_ and (C) Glc_6_ as the substrates.

Although the activity of *Ml*GH17B on Glc_4_ was negligible compared to the activity on Glc_5_ and Glc_6_, product spectra detected by MALDI-TOF, after 48 h incubation (Fig. 5A), demonstrated four predominant quasi-molecular ions [M + K]^+^ at *m/z* 705.24, *m/z* 1029.4, *m/z* 1191.4, and *m/z* 1515.5 corresponding to the substrate Glc_4_ and the transferase products Glc_6_, Glc_7_, and Glc_9_ respectively. Additionally, a peak denoting Glc_10_ was observed with quasi-molecular ions [M + K]^+^ at *m/z* 1677.6, albeit less pronounced. Moreover, minor peaks associated with quasi-molecular ion [M + K]^+^ at *m/z* 867.5, *m/z* 1353.5, *m/z* 1839.7 and *m/z* 2001.7 representing Glc_5_, Glc_8_, Glc_11_, and Glc_12_ were also detected.

The MS data showed a substantial quantity of unexpected products using Glc_4_ as substrate, such as Glc_7_ and Glc_9_, which were generated during the course of the reaction. As Glc_4_ cannot occupy all putative five binding sites in the enzyme, it is likely that it binds in two alternative positions, specifically in the −3 to +1 subsites, as well as in the −2 to +2 subsites that is required for cleavage of Glc_2_. This may result in the cleavage of only one glucose molecule from the reducing end, instead of the predominantly formed Glc_2_. This alternative subsite binding could subsequently be confirmed by HPAEC-PAD product profile analysis in which a small peak corresponding to Glc was detected after 24 h incubation (Fig. 4). A DP4-oligo acceptor molecule will then enable the creation of a β-1,6-linkage, leading to the production of Glc_7_.

Following 48 h incubation with Glc_5_, MALDI-TOF mass spectra (Fig. 5B) displayed four major quasi-molecular ions [M + K]^+^ at *m/z* 867.2, *m/z* 1353.3, *m/z* 1839.5, and *m/z* 2325.8 corresponding to Glc_5_, Glc_8_, Glc_11_ and Glc_14_. The minor presence of Glc_4_ at *m/z* 705.14 can be attributed to a slight contamination of the Glc_5_ substrate. The transferase products derived from Glc_5_ exhibited a three glucose unit interval, denoting the bound donor, in which Glc_8_ was the first major product, where Glc_5_ served as acceptor. Lesser peaks at *m/z* 2812.4 and *m/z* 3301 corresponding to quasi-molecular ion [M + K]^+^ of Glc_17_ and Glc_20_ were also observed. The minor peaks at *m/z* 1029 and *m/z* 1191.4, representing quasi-molecular ion [M + K]^+^ of Glc_6_ and Glc_7_ could be transferase product when Glc_4_ acted as donor and/or acceptor substrate, respectively.

A similar pattern was observed when the enzyme was incubated with Glc_6_ (Fig. 5C). The MALDI-TOF product profile of *Ml*GH17B after 48 h incubation with Glc_6_ illustrated four primary quasi-molecular ions [M + K]^+^ at *m/z* 1029.3, *m/z* 1677.6, *m/z* 2326.1, and *m/z* 2974.8 corresponding to Glc_6_, Glc_10_, Glc_14,_ and Glc_18_, confirming the cleavage of Glc_2_ and transferring the remaining donor part to another molecule of substrate. Nevertheless, minor peaks indicative of Glc_22_ with quasi-molecular ion [M + K]^+^ at *m/z* 3623.8 and Glc_26_ with quasi-molecular ion [M + K]^+^ at *m/z* 4271.8 were also detected. This long incubation time resulted in occurrence of a peak at *m/z* 705.3, corresponding to quasi-molecular ion [M + K]^+^ of Glc_4_, which is the product of the cleavage, and a peak at *m/z* 2164 corresponding to quasi-molecular ion [M + K]^+^ of Glc_13_.

### NMR studies of the transferase product of *Ml*GH17B

**Fig. 6:**
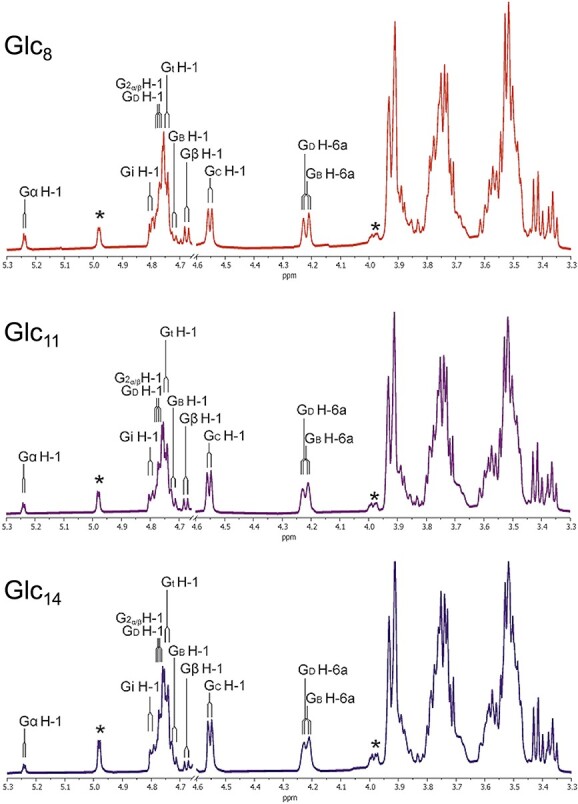
One-dimensional ^1^H-NMR spectra of Glc_8_, Glc_11_, and Glc_14_ reaction products of *Ml*GH17B using laminaripentaose (Glc_5_) as the substrate at activity optimum conditions for 6 h, recorded at D_2_O, 311 K. ^*^outlines minor contamination of dextran in the samples. The possible linkages are shown as Gt [β-d-Glc*p*-(1 → 3)-]; Gi [-(1 → 3)-β-d-Glc*p*-(1 → 3)-]; G2α/β (next to reducing end) [-(1 → 3)-β-d-Glc*p*-(1 → 3)-]; Gα/β (reducing end) [-(1 → 3)-β-d-Glc*p*]; GB [-(1 → 6)-β-D-Glc*p*-(1 → 3)-]; Gc [-(1 → 3)-β-d-Glc*p*-(1 → 6)-]; and Gd [-(1 → 3,6)-β-d-Glc*p*-(1 → 3)-].

**Fig. 7:**
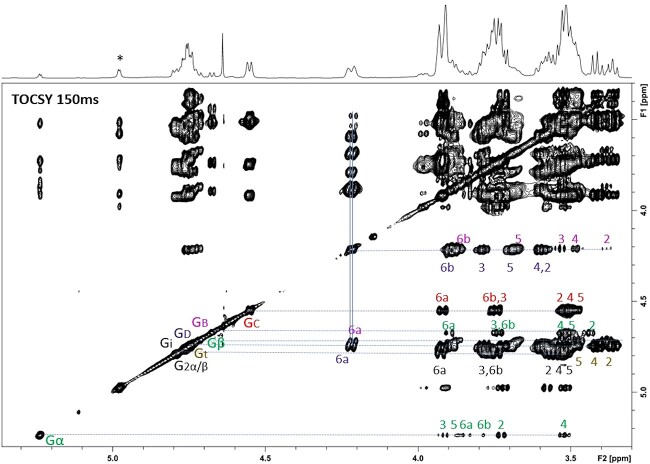
The 150 ms TOCSY spectra of Glc_8_, Glc_11_, and Glc_14_ reaction products of *Ml*GH17B using laminaripentaose (Glc_5_) as the substrate at activity optimum conditions for 6 h. The possible linkages are shown as Gt [β-d-Glc*p*-(1 → 3)-]; Gi [-(1 → 3)-β-d-Glc*p*-(1 → 3)-]; G2α/β (next to reducing end) [-(1 → 3)-β-d-Glc*p*-(1 → 3)-]; Gα/β (reducing end) [-(1 → 3)-β-d-Glc*p*]; GB [-(1 → 6)-β-D-Glc*p*-(1 → 3)-]; Gc [-(1 → 3)-β-d-Glc*p*-(1 → 6)-]; and Gd [-(1 → 3,6)-β-d-Glc*p*-(1 → 3)-].

**Fig. 8:**
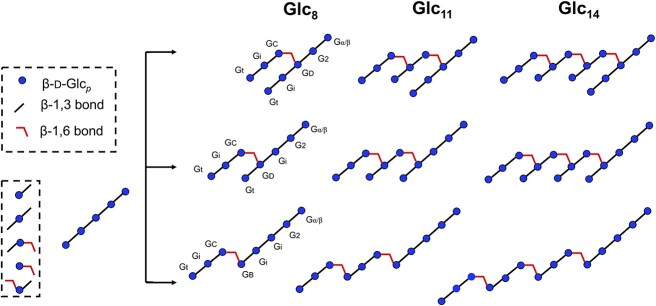
Proposed structure for transglycosylation products of *Ml*GH17B acting on laminaripentaose (Glc_5_) substrate. Glc_8_, Glc_11_, and Glc_14_ represent the gluco-oligosaccharides with 8, 11, and 14 glucose units. Gα/β represents the reducing end. The possible linkages are shown as Gt [β-d-Glc*p*-(1 → 3)-]; Gi [-(1 → 3)-β-d-Glc*p*-(1 → 3)-]; G2 (next to reducing end) [-(1 → 3)-β-d-Glc*p*-(1 → 3)-]; Gα/β (reducing end) [-(1 → 3)-β-d-Glc*p*]; GB [-(1 → 6)-β-D-Glc*p*-(1 → 3)-]; Gc [-(1 → 3)-β-d-Glc*p*-(1 → 6)-]; and Gd [-(1 → 3,6)-β-d-Glc*p*-(1 → 3)-]. The nomenclature of symbols follows the symbol nomenclature for glycans (SNFG) published by Neelamegham et al. 2019 (Neelamegham et al. 2019).

The one-dimensional ^1^H spectra of purified transglycosylation products, Glc_8_, Glc_11_, and Glc_14_ were very similar (Fig. 6). The one-dimensional ^1^H NMR spectra of Glc_8_ combined with the two-dimensional total correlation spectroscopy (TOCSY) (150 ms) (Fig. 7) and heteronuclear single quantum coherence (HSQC) measurements (spectra not shown) of Glc_8_, showed anomeric signals for β-D-Glc*p*-(1 → 3)- (Gt, δ 4.75); -(1 → 3)-β-D-Glc*p*-(1 → 3)- (Gi, δ 4.79); -(1 → 3)-β-D-Glc*p*-(1 → 3)- [G2α/β, next to reducing-end Gα/β, δ 4.75 (H-1α), δ 4.77 (H-1β)]; -(1 → 3)-D-Glc*p* [Gα/β, δ 5.24 (H-1α), δ 4.68 (H-1β)]; -(1 → 3)-β-D-Glc*p*-(1 → 6)- (GC, δ 4.55); -(1 → 3,6)-β-D-Glc*p*-(1 → 3)- (GD, δ 4.78); and -(1 → 6)-β-D-Glc*p*-(1 → 3)- (GB, δ 4.76) residues as Glc*p* stands for glucopyranose (Table 2) (Hreggvidsson et al. 2011; Dobruchowska et al. 2016). The complete sets of proton signals of the GB and GD residues were established by their specific H-2,3,4,5,6a,6b signal sets found via GB H-6a (δ 4.21) and GD H-6a (δ 4.22) tracs (Fig. 7). The HSQC spectrum (data not shown) demonstrated a downfield shift for GB and GD C-6 (δC-6 69.7), indicating the involvement of C-6 in a glycosidic linkage. The presence of GB and GD residues suggest that Glc_8_, Glc_11_, and Glc_14_ compounds contain single, double, and triple branching (branching; residue GD), or/and single, double, and triple kinks (elongation; residue GB). A total elongation/branching degree was determined from the ratio of the internal –(1 → 6)-β-D-Glc*p*-(1 → 3)- (GB) H-1 and branched –(1 → 3,6)-β-D-Glc*p*-(1 → 3)- unit (GD) H-6a proton area to that of H-1 of the reducing Gα/β residue in the one-dimensional ^1^H NMR spectra of Glc_8_, Glc_11_, and Glc_14_ (Fig. 6), revealing that the trans-β-glucosidase reaction was estimated at 70% inside the acceptor chain and 30% at the non-reducing end of the acceptor using Glc_5_ as substrate. Based on the collected NMR data, including the anomeric signal peak area ratio Gα/β: GC:(GB,GD,Gi,Gt,G2α/β) = 1:1:6 for Glc_8_, Gα/β:GC:(GB,GD,Gi,Gt,G2α/β) = 1:2:8 for Glc_11_, and Gα/β:GC:(GB,GD,Gi,Gt,G2α/β) = 1:3:10, it is assumed that the Glc_8_, Glc_11_, and Glc_14_ isomeric structures are synthesized by a transfer of a β-D-Glc*p*-(1 → 3)-β-D-Glc*p*-(1 → 3)-β-D-Glc*p* trisaccharide unit from the non-reducing end of a donor Glc_5_ substrate to the terminal non-reducing end or the second and third residues (from the non-reducing end) of the acceptor Glc_5_ substrate via an O − 3 → O-6 transition (Glc_8_), followed by the second and third addition of a β-D-Glc*p*-(1 → 3)-β-D-Glc*p*-(1 → 3)-β-D-Glc*p*- trisaccharide unit (Glc_11_ and Glc_14_). The NMR spectroscopy resulted in schematic comprehension of the structure of transglycosylation products (Fig. 8).

**Table 2:** ^1^H and ^13^C NMR chemical shifts (δ) of reaction products obtained after incubation of laminari-oligosaccharides with *Ml*GH17B enzyme, recorded in D_2_O at 311 K.

	**Residue**	**H-1** **C-1**	**H − 2** **C-2**	**H-3** **C-3**	**H-4** **C-4**	**H-5** **C-5**	**H-6a/6b** **C-6**
**Gα**	-(1 *→* 3)-α-D-Glc*p*	5.24 93.0	3.73 72.0	3.92 83.3	3.52 70.0	3.86 72.2	3.83/3.79 61.6
**Gβ**	-(1 *→* 3)-β-D-Glc*p*	4.68 96.6	3.44 74.9	3.74 85.7	3.52 69.2	3.49 76.6	3.90/3.74 61.6
**G2α**	-(1 → 3)-β-D-Glc*p*-(1 → 3)-α-D-Glc*p*	4.75 103.7	3.56 74.3	3.78 85.3	3.54 69.1	3.52 76.7	3.93/3.74 61.7
**G2β**	-(1 → 3)-β-D-Glc*p*-(1 → 3)-β-D-Glc*p*	4.77 103.7	3.56 74.3	3.78 85.3	3.53 69.1	3.53 76.7	3.93/3.74 61.7
**Gi**	-(1 → 3)-β-D-Glc*p*-(1 → 3)-	4.79 103.4	3.56 73.9	3.77 85.5	3.54 69.1	3.52 76.6	3.92/3.76 61.7
**Gt**	β-D-Glc*p*-(1 → 3)-	4.75 103.7	3.37 74.4	3.53 76.6	3.42 70.6	3.48 76.8	3.92/3.72 61.7
**GB**	-(1 → 6)-β-D-Glc*p*-(1 → 3)-	4.76 103.8	3.38 74.4	3.53 76.5	3.48 70.5	3.67 75.6	4.21/3.87 69.7
**GC**	-(1 → 3)-β-D-Glc*p*-(1 → 6)-	4.55 103.6	3.52 73.6	3.74 85.6	3.51 69.1	3.50 76.6	3.92/3.75 61.6
**GD**	-(1 → 3,6)-β-D-Glc*p*-(1 → 3)-	4.78 103.8	3.60 74.0	3.79 85.4	3.58 69.1	3.69 75.4	4.22/3.89 69.7

The NMR analysis indicated that Glc_7_ likely forms following the binding of the donor substrate to subsites −3 to +1, with Glc acting as a leaving group. The presence of a laminaritetraose (Glc_4_) acceptor molecule then facilitates the formation of a β-1,6-linkage, resulting in the production of Glc_7_. This unexpected product pattern, observed in the presence of Glc_4_, could be due to the substrate not occupying all the enzyme’s subsites. This allows for two alternative positions (as previously mentioned), demonstrating that binding at either subsite −3 or +2 can be favored (Supplementary Fig. S4).

### Three-dimensional structure model and validation

**Fig. 9:**
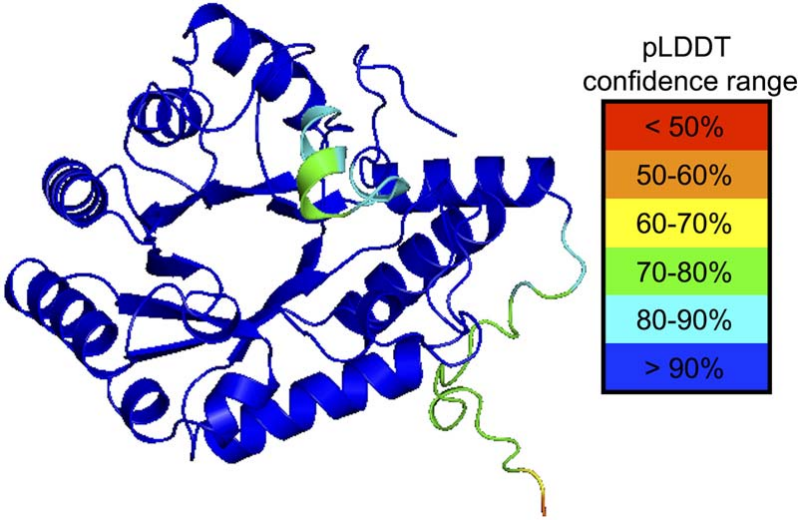
*Ml*GH17B modeled by AlphaFold2 with pLDDT confidence levels mapped onto the structure.

To date, only one crystal structure of a GH17 transglycosylase is solved, which is the structure of the fungal enzyme *Rm*Bgt17A from *R. miehei* (Qin et al. 2015). Homology models are, however, available for three bacterial transglycosylases: Glt1 from *P. aeruginosa*, Glt3 from *P. putida,* and Glt20 from *B, diazoefficien*s (Linares-Pastén et al. 2021). Due to the low number of available structures, *Ml*GH17B was modeled using AlphaFold2 (Jumper et al. 2021; Mirdita et al. 2022) using the Colab notebook to determine the structural organization (Fig. 9).

The predicted three-dimensional model of the enzyme, generated by AlphaFold2 indicated that the enzyme has a TIM-barrel ((β/α)_8_) fold that spans the 295 amino acids. A significant portion of this model was characterized by a high Predicted Local Distance Difference Test (pLDDT) score, exceeding 90%. Two segments of the model displayed a moderate pLDDT score: the N-terminal segment (first 18 amino acid residues) and the region from Ser270 to Gly280 (SWKVGSEGDVG) corresponding to η6-α8-η7 (Fig. 9 and Supplementary Fig. S5), with pLDDT scores ranging from 70 to 90%. Moreover, the predicted position of Glu34 was categorized with a moderate confidence level, falling between 80–90% (these regions are shown in red boxes in Supplementary Fig. S5).

**Fig. 10:**
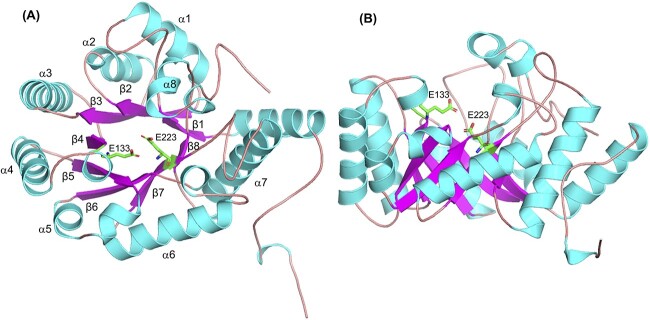
Modeled structure of *Ml*GH17B from AlphaFold2, represented as a ribbon diagram. The TIM-barrel ((β/α)_8_) structure of the enzyme is illustrated from (A) the front view and from (B) the side view. The catalytic proton donor (Glu133) and nucleophile (Glu223) residues are indicated in the model.

The conserved catalytic residues, Glu133 and Glu223 (shown in Fig. 10), were located at about two-thirds along the length of the catalytic cleft at an inter-residue distance of 5 Å. The length and geometry of the catalytic cleft makes it possible for the enzymes to accommodate longer-chain oligosaccharides and laminarin (as confirmed by the activity determinations).

A hybrid homology model was also generated in the YASARA program with the crystal structures of *Rm*Bgt17A from *R. miehei* (PDB 4WTP, 4WTR, and 4WTS, 28.73% sequence identity) and the laminarinase *Fb*GH17A from *Formosa* sp. (PDB 6FCG) with sequence similarity 21.92% selected as main templates to create different fragments of the model (Table S1). The model’s quality was assessed through ERRAT, VERIFY3D, and PROCKECK using the UCLA–DOE LAB–SAVES v6.0 server (saves.mbi.ucla.edu). The results indicated that the YASARA-generated model exhibited satisfactory quality. Further details on the model’s quality assessment can be found in the supplementary materials, specifically in the “Quality assessment of YASARA three-dimensional model” section. Nevertheless, a comparison with the AlphaFold2 model revealed variations in the size and orientation of secondary structures’ elements, particularly in the loops surrounding the active site (Supplementary Fig. S5). Subsequently, the AlphaFold2 model was employed for further investigations involving the docking of Glc_5_ to the enzyme’s active site.

**Fig. 11:**
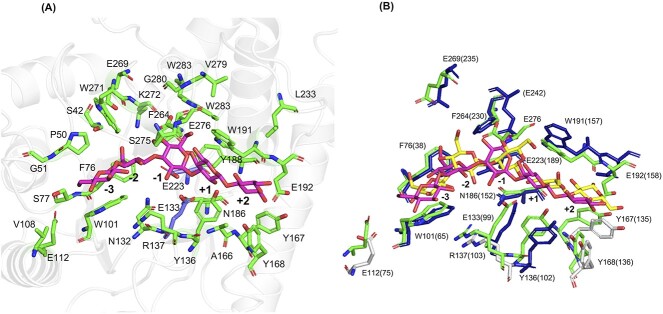
Active site architecture of *Ml*GH17B. A) the residues within 5 Å of Glc_5_ located from −3 to +2 subsites of *Ml*GH17B; conserved proton donor and nucleophile residues are outlined in purple, and the ligand is shown in magenta. B) Conserved residues between *Ml*GH17B (green) and *Rm*Bgt17A (dark blue), the ligand belonging to *Ml*GH17B *and Rm*Bgt17A are shown in magenta and yellow, respectively; conserved residues, which do not participate in interactions within the active site of *Rm*Bgt17A, are represented in silver.

The docking of laminaripentaose (Glc_5_) was carried out using local docking with AutoDock, which is implemented into YASARA. To evaluate the quality of the resulting docked model, it was compared to the structure of *Rm*Bgt17A co-crystallized with laminaribiose (PDB 4WTR) and laminaritriose (PDB 4WTS) (Fig. 11). This comparison involved assessing the alignment of the ligand, its conformation, glycosidic linkages within the sugar units, as well as the interactions between the ligand and the enzyme.

### Superimposition, docking and three-dimensional structure analysis

Structural superimposition of *Ml*GH17B with *Rm*Bgt17A, Glt1, Glt3, and Glt20 (as well as docking experiments) demonstrated that despite a low sequence similarity, *Ml*GH17B, Glt20, and *Rm*Bgt17A, shared an overall similar active site architecture, with the exception that *Ml*GH17B and *Rm*Bgt17A has five subsites consisting of three glycone subsites (−3, −2, and −1) interacting with the donor and two aglycone subsites (+1, +2) interacting with the acceptor, while the active site of Glt20 is comprised of six potential subsites, with one additional glycone subsite (−4, −3, −2, −1, +1, +2) (Linares-Pastén et al. 2021).

*Ml*GH17B was lacking the additional subdomain (characteristic for plant endo-β-glucanases in GH17) located at the end of the catalytic cleft in the aglycone region. This subdomain has been associated with the extension of the aglycone subsites in the eukaryotic plant enzymes. Qin et al. (Qin et al. 2015) demonstrated that the fungal transglycosylase *Rm*Bgt17A lacks this subdomain, and Linares-Pastén et al. (Linares-Pastén et al. 2021) confirmed that proteobacterial enzymes (Glt1, Glt3, and Glt20) also lack this subdomain, indicating that this may play a role for transglycosylation. However, structural models, generated for bacterial GH17 endo-β-glucanases using Alphafold2 in this study (supplementary Fig. S6), including *Fa*GH17A (GenBank CDF79584.1) from *Formosa agariphila*, *Fb*GH17B (GenBank AOR29491.1) from *Formosa* sp. Hel1_33_131, *Vb*GH17A (GenBank OEF87991.1) from *Vibrio breoganii*, *Vv*GH17 (GenBank ASM98089.1) from *Vibrio vulnificus*, as well as the crystal structure of *Fb*GH17A (GenBank AOR29489.1), from *Formosa* sp. Hel1_33_131 consistently revealed absence of the subdomain at the aglycone part of the active site. As a consequence, all bacterial GH17 enzymes encompassing both laminarinases and transglycosylases (including *Ml*GH17B) exhibit shorter active sites within the aglycone region in comparison to the eukaryotic plant endo-β-glucanases.

The active site of *Ml*GH17B is surrounded by 8 loops, which may influence the temperature optimum for activity, while the core of the *Ml*GH17B exhibits higher temperature stability according to DSF as the T_m_ of the protein is significantly higher than the activity optimum. Subsite −3 is surrounded by loops β1–α1, β2–α2 and β3–α3; subsites −2 is surrounded by the β8–α8 loop; subsite −1 is surrounded by the β4–α4, β7–α7 and β8–α8 loops; subsite +1 is surrounded by loops β4–α4, and β6–α6, and subsite +2 is surrounded by loops β5–α5 and β6–α6, with the proton donor (Glu133) and nucleophile (Glu223) residues located in loop β4–α4 and at the C-terminal of β7, respectively.

**Fig. 12:**
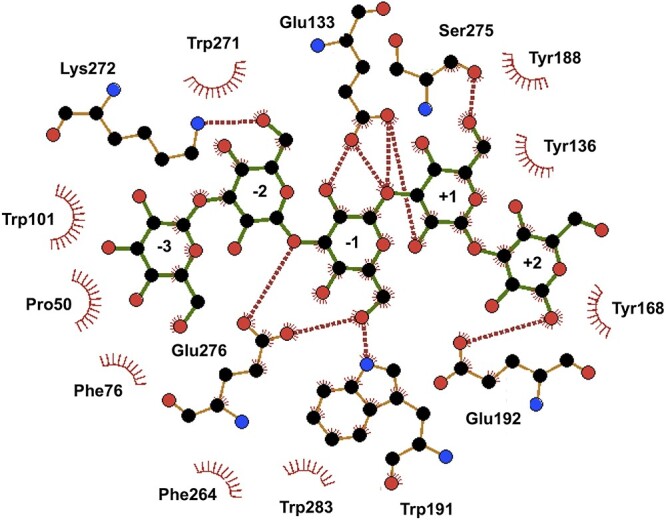
Glucan interaction in *Ml*GH17B. Interactions with the sugar residues from Glc_5_ substrate bound in subsites −3 to +2 of *Ml*GH17B, shown using LigPlot^+^ (Laskowski and Swindells 2011).

The potential substrate interacting residues, were indicated as those located within 5 Å around the Glc_5_ ligand (Fig. 11A) with conserved positions of interacting residues between *Ml*GH17B and *Rm*Bgt17A from *R. miehei* (Fig. 11B) which was co-crystallized with laminaribiose (PDB 4WTR) and laminaritriose (PDB 4WTS) (Qin et al. 2015). Active site comparison between *Ml*GH17B and *Rm*Bgt17A (Fig. 11B) revealed 13 conserved residues between the enzymes, excluding the proton donor (Glu133 in *Ml*GH17B and Glu99 in *Rm*Bgt17A) and nucleophile (Glu233 in *Ml*GH17B and Glu189 in *Rm*Bgt17A) residues. Among these residues, Glu112 (Glu75 in *Rm*Bgt17A), Arg137 (Arg103 in *Rm*Bgt17A), Tyr167 (Tyr135 in *Rm*Bgt17A) and Tyr168 (Tyr136 in *Rm*Bgt17A) are conserved between both enzymes and occupy corresponding positions in the superimposed structures (Fig. 11B), indicative of common functions, although these residues at corresponding positions, were not reported by Qin et al. (Qin et al. 2015) as directly involved in the hydrolysis or transglycosylation events of *Rm*Bgt17A. The potential ligand interactions between *Ml*GH17B and the Glc_5_ ligand are visually presented in Fig. 12 and detailed in Table 3, while a comparison of the interactions in *Ml*GH17B and *Rm*Bgt17A is shown in Table 4.

**Table 3:** Interactions between *Ml*GH17B and the Glc_5_ ligand bound in subsites −3 to +2, obtained using LigPlot^+^.

**Subsite**	**Saccharide atom**	**H-bond distance**	**Residue atom**	**Interacting residue**
−**3**		Stacking		Pro50
		Stacking		Phe76
		Stacking		Trp101
−**2**	O6	3.19	NZ	Lys272
		Stacking		Trp271
		Stacking		Phe264
**−1**	O2	2.76	OE1	Glu133
	O6	3.00	NE1	Trp191
	O6	3.21	OE1	Glu276
	O3	3.26	OE2	Glu276
		Stacking		Trp283
**+1**	O3	2.86	OE1	Glu133
	O2	3.22	OE2	Glu133
	O3	3.04	OE2	Glu133
	O6	2.7	O	Ser275
		Stacking		Tyr188
**+2**	O1	2.85	OE2	Glu192
		Stacking		Tyr136
		Stacking		Tyr168

**Table 4:** Interaction of the sugar residues of Glc_5_ and the active site residues of *Ml*GH17B and *Rm*Bgt17A.

Subsites	*Rm*Bgt17A		*Ml*GH17B	
	Residue	Saccharide atom	Residue	Saccharide atom
**Conserved residues**				
−**2**	Glu235	OH6	Glu269	-
−**1**	Glu242	OH4	Glu276	OH3/OH6
	Glu99	OH1	Glu133	OH2
	Asn152	OH1	Asn186	-
	Trp157	OH6	Trp191	OH6
**+1**	Glu99	OH2	Glu133	OH2/OH3
**+2**	Glu158	OH1	Glu192	OH1
**Non-conserved residues**				
**−3**	ASP67^a^	OH3	No aligned residue	-
**−2**	Val10^a^	OH4	No aligned residue	-
	Arg238	O5	Lys272	OH6
**−1**	Arg238	OH4	Lys272	-
	Ser98^a^	OH2	Asn132	-
**+1**	Val241	-	Ser275	OH6
**+2**	pHe154	OH1/OH2	Tyr188	-

^a^Residues are involved in water mediated hydrogen bound.

At the glycone subsites, Phe76 (Phe38 in *Rm*Bgt17A), Trp101 (Trp65 in *Rm*Bgt17A), Glu269 (Glu235 in *Rm*Bgt17A), and Phe264 (Phe230 in *Rm*Bgt17A) are conserved and superimpose well. Glu276 (Glu242 in *Rm*Bgt17A) is also conserved but did not superimpose with the residue in *Rm*Bgt17A. The position of Glu276, however, falls within a region of the model with a pLDDT score ranging from 70 to 90%. The variation in the structural position of Glu276 compared to Glu242 in *Rm*Bgt17A could be attributed to the model’s lower confidence in this specific region (Fig. 9 and Supplementary Fig. S5). In the glycone region, Asn132 and Lys272 in *Ml*GH17B were replaced by Ser98 and Arg238 in *Rm*Bgt17A, respectively (Fig. 11). At subsites −2 and −3, aromatic residues including Phe76 (Phe38 in *Rm*Bgt17A), Trp101 (Trp65 in *Rm*Bgt17A), and Trp271 stacked against the glucose units in the docked ligand, forming a hydrophobic sugar-binding platform. Potential hydrogen bonds were observed between Lys272 and OH6 of the glucose in −2 glycone subsite, and between Glu276 and OH3 and OH6 of the glucose in −1 subsite. Moreover, Glu133 potentially forms hydrogen bonds with OH2 in the −1 subsite, and Trp191 could potentially form a hydrogen bond with OH6 in the same subsite.

The conserved residues in the aglycone subsites also superimpose well in the two structures (Fig. 11B). Three residues, Trp191 (Trp157 in *Rm*Bgt17A), Glu192 (Glu158 in *Rm*Bgt17A) and Tyr136 (Tyr102 in *Rm*Bgt17A) are conserved and reported to play an important role in the transglycosylation transition and β-1,6-linkage formation (Qin et al. 2015). Moreover, Asn186 (Asn152 in *Rm*Bgt17A) is also conserved between *Ml*GH17B and *Rm*Bgt17A. However, Phe154 in *Rm*Bgt17A is replaced by Tyr188 in *Ml*GH17B. The residues Tyr167 (Try135 in *Rm*Bgt17A), Tyr168 (Tyr136 in *Rm*Bgt17A), and Glu192 (Glu158 in *Rm*Bgt17A) were found near the reducing end of the glycosyl at the +2 subsite (Fig. 11A and B), and potentially constrain both the entrance position and the orientation of the acceptor substrate within the catalytic cleft. Potential hydrogen bonds were observed between Glu192 and OH1 of the glucose moiety in the +2 aglycone subsite, between Ser275 and OH6 of the glucose moiety in the +1 subsite, and between the catalytic residue Glu133 with OH2 and OH3 of the glucose moiety in +1 subsite.

As evident from the data presented in Table 4, there is a notable conservation of hydrogen bond interactions between *Ml*GH17B and *Rm*Bgt17A within the −1, +1, and +2 subsites. Many of these interactions were recognized as crucial for the hydrolysis and transglycosylation activities of *Rm*Bgt17A (Qin et al. 2015). Specifically, the hydrogen bond interaction involving Trp191 (Trp157 in *Rm*Bgt17A) with OH6 of the glucose moiety in subsite −1 assumes a critical role in stabilizing the transition state for both transglycosylation and hydrolysis reactions. Additionally, Trp191 (Trp157 in *Rm*Bgt17A) and Tyr136 (Tyr102 in *Rm*Bgt17A) serve as essential sugar-binding platforms within the +1 subsite. Furthermore, Glu192 (Glu158 in *Rm*Bgt17A) exhibits a conservation of hydrogen bond formation with OH1 of the glucose moiety in the +2 subsite, enabling it to establish direct hydrogen bonds with the acceptor substrate within the +2 subsite for the transglycosylation reaction (Qin et al. 2015).

## Discussion

This study provides detailed insights into the structure and function of the GH17 transglycosylase *Ml*GH17B of marine origin which is the first transglycosylase from this environment to be characterized. The enzyme is encoded in the genome of the marine bacterium *Muricauda lutaonensis* strain ISCAR-4703, and related candidates has in this study been discovered, encoded in the genomes of other *Muricauda* species, suggesting that the enzyme likely plays a significant role in the degradation and/or storage of laminarin in these related marine microorganisms. Moreover, sequence similarity analysis using blastp, demonstrated presence of numerous homologs in deposited genomes of other marine bacteria, indicating this to be a common type of activity in the marine environment. This may not be surprising as laminarin is a common carbohydrate polymer in different marine brown algal species (Hreggviðsson et al. 2020). Currently, the genus *Formosa*, contains the more investigated bacterial species concerning characterization of carbohydrate converting enzymes active against seaweed polymers. However, no enzyme has yet been characterized as a GH17 transglycosylase from any species of this genus, although the catalytic module of the exoglucanase *Fb*GH17B from *Formosa* sp. Hel1_33_13 that is releasing glucose from the reducing end of laminarin (Unfried et al. 2018), is sharing 53% sequence identity with *Ml*GH17B. A difference is that *Fb*GH17B is connected to a membrane transporter, but whether that has implications on its activity is not known. The homology to *Ml*GH17B, however makes it possible that *Fb*GH17B may exhibit a yet undetected transglycosylase activity or that this is missing due to missing crucial residues important for transglycosylation, as discussed below.

The to date characterized GH17 bacterial transglycosylases all originate from soil environments. Glt1 from *Pseudomonas aeruginosa*, Glt3 from *Pseudomonas putida*, Glt7 from *Azotobacter vinelandii*, all originate from soil bacteria. Opposed to *Ml*GH17B, these enzymes cleave the substrate from the non-reducing end during the first step and then, dependent on enzyme, perform transglycosylation activities such as β-1,3-elongation, β-1,4- or β-1,6- elongation and β-1,6- branching (Hreggvidsson et al. 2011). Only Glt20 from the soil bacterium *B. diazoefficiens* (Linares-Pastén et al. 2021) and *Rm*Bgt17A from the compost thriving fungus *R. miehei* (Qin et al. 2015), show transglycosylation activity resembling that of *Ml*GH17B: Cleaving the substrate from the reducing end, producing Glc_2_, and transferring the remaining part of the donor substrate to the non-reducing end of the acceptor molecule (Linares-Pastén et al. 2021). *Ml*GH17B is in this work shown to be structurally homologous to *Rm*Bgt17A, with a similar number of subsites (−3, −2, − 1, +1, +2), while the modeled structure of Glt20 is predicted to have an additional −4 glycone subsite. The high resemblance between *Ml*GH17B and *Rm*Bgt17A shows that enzymes of similar structures have been adapted to participate in conversions of β-1,3-linked glucans from their respective habitat.

*Ml*GH17B has a distinct specificity toward β-1,3-linkages, and a minimum substrate length of DP4 (confirmed using Glc_4_ as substrate) which, however, displays low activity compared to Glc_5_ and longer substrates where all five subsites in the catalytic cleft are filled. Activity on polymeric laminarin proves that the substrate chain is allowed to extend beyond subsite −3, but with reduced specific activity compared to the activity on laminari-oligosaccharides (DP5 or DP6). Steric hindrance by the MBP, negatively affecting binding of larger substrate molecules to the enzyme, can however not be excluded at this stage.

*Rm*Bgt17A and *Ml*GH17B shared 13 potential substrate binding residues (excluding the two catalytic residues Glu133 and Glu233) over five subsites, corroborating similar functions. HPAEC-PAD, MALDI-TOF, and NMR analysis unambiguously showed that *Ml*GH17B cleaved off two residues from the reducing sugar end (the aglycone subsites +1 and +2), in principal regardless of the substrate size (the only exception being Glc_4_ where small amounts of glucose were released), followed by transfer of the remaining part to an acceptor substrate molecule, generating a β-1,6-linkage. Among the conserved substrate binding residues, those in the aglycone subsites, have been proposed to be of special importance for the transglycosylation transition, especially the three residues, Trp191 (Trp157 in *Rm*Bgt17A), Glu192 (Glu158 in *Rm*Bgt17A) and Tyr136 (Tyr102 in *Rm*Bgt17A) (Qin et al. 2015). The corresponding residues have been mutated to Ala in *Rm*Bgt17A (Tyr102Ala, Trp157Ala and Glu158Ala) resulting in completely abolished transglycosylation ability of the enzyme (Qin et al. 2015), while approximately 14% of the transglycosylation activity was retained when Trp was replaced with the smaller aromatic residue Phe (Trp157Phe) (Qin et al. 2015). Trp191 is proposed to both hydrogen bond with OH6 of the glucose in the −1 subsite (stabilizing the transition state) and together with Tyr136 serve as a sugar-binding platform within the +1 subsite, while Glu192 is proposed to hydrogen bond with OH1 in the +2 subsite. The presence of Tyr136, Trp191, and Glu192 in *Ml*GH17B thus leads to the possibility of the enzyme to bind acceptor molecules (>DP2) in an alternate position, possible when the donor is already bound in the catalytic cleft of the enzyme, in line with the proposed binding in *Rm*Bgt17A. Binding of the acceptor has been proposed to occur on top of the catalytic site, which would promote β-1,6-linkage specificity (Qin et al. 2015) based on the structure of the laminari-oligosaccharide. These findings underscore the pivotal role played by rather few residues in enhancing the enzyme’s transglycosylation activity. Their absence may also serve as an alternative explanation for lack of transglycosylation in otherwise related enzymes, such as *Fb*GH17B. The alignment (Fig. 2) for example shows that Tyr136 (Tyr102 in *Rm*Bgt17A) is replaced by a Met in *Fb*GH17B, while the residues corresponding to Trp191 and Glu192 (*Ml*GH17B numbering) are conserved.

The high number of conserved residues in subsites −1, +1 and +2 subsites, combined with the similarities in the observed transglycosylation pattern, makes it likely that *Ml*GH17B and *Rm*Bgt17A share the same two-step catalytic mechanism as proposed by Qin et al. (Qin et al. 2015). In the first step, the donor substrate (laminari-oligosaccharide) occupies the active site (−3 to +2 subsites) of the enzyme and the departure of the Glc_2_ leaving group (from subsites +1 and +2) is mediated by the proton donor (Glu133) donating a proton to the O atom between the glycosyl moieties located at sites −1 and +1, while the nucleophile Glu223 forms a covalent intermediate with the remaining part of the donor substrate. In the second step, the acceptor substrate molecule occupies the +1 and +2 subsites, in a different way than the original substrate, promoting subsequent formation of the β-1,6-linkage. The deprotonated proton donor (Glu133) activates the C-6 hydroxyl of a glucose moiety of the acceptor, which carries out a nucleophilic attack on the enzyme-substrate intermediate complex, leading to formation of a transglycosylation product with a β-1,6-linkage. No potential hydrogen bonds were detected between Glu233 and the substrate in the current model. It is, however, noteworthy that the docking simulations conducted in this study do not account for the presence of structural water molecules within the catalytic cleft. In the crystal structure of *Rm*Bgt17A, three water molecules have been identified (Table 4), which increased the number of hydrogen bonds formed between the ligand and active site residues of the enzyme (Qin et al. 2015).

In both *Ml*GH17B and *Rm*Bgt17A, the reducing end of the donor substrate is always bound in subsite +2. In addition, the −3 subsite must be important for binding and activity of the enzyme, as Glc_4_ was a relatively poor substrate. In the case of Glc_4_, the substrate could, based on the collected data, bind in alternative positions, either in the −3 to +1 subsites or in the −2 to +2 subsites, resulting in either Glc or Glc_2_ as leaving groups. The NMR-data revealed that a β-1,6-linkage is formed between the glucose moiety of the donor substrate bound in the −1 subsite and either the non-reducing end sugar unit of the acceptor (creating a kink) or the second or third glucose from the non-reducing end in the acceptor molecule (creating a branch). This will lead to synthesis of oligosaccharides with either branched or kinked structure.

Comparison of the modeled structure of *Ml*GH17B with the corresponding structure of *Rm*Bgt17A (Qin et al. 2015) showed similarities in catalytic cleft architecture, specifically in subsites −1, +1 and +2. In *Ml*GH17B, the three glycone subsites are surrounded by polar residues, as was also observed for *Rm*bgt17A, however, several aromatic residues in the glycone subsites, namely Phe76, Trp101, Phe264, Trp271 and Trp283 are involved in hydrophobic interactions with the donor substrate. The two aglycone subsites are surrounded by hydrophobic residues providing a hydrophobic sugar binding platform, which could be the reason for the release of Glc_2_ from the reducing end while the rest of the substrate remains bound in the active site for the transfer reaction. The MALDI-TOF mass spectrometry results also demonstrated the ability of *Ml*GH17B to synthesize long-chain, multiple branched, oligosaccharides such as Glc_20_ and Glc_26_ from Glc_5_ and Glc_6_ substrates, respectively, using long reaction time, allowing us to conclude that branched oligosaccharides (dependent on their respective concentration) can also be either donors or acceptors in the transglycosylation reaction.

## Conclusion

In this study, a novel β-1,3-glucanosyltransglycosylase *Ml*GH17B from the marine *Muricauda lutaonensis* strain ISCAR-4703 was characterized in terms of substrate specify and product profile. DSF analysis showed that the enzyme displayed a thermostable core, while being active at lower temperature, a feature that may be beneficial at geothermal marine sites, where temperature gradients can be expected. The three-dimensional structure of the enzyme was modeled and Glc_5_ was docked into the catalytic cleft revealing five potential subsites (−3, −2, −1, +1, +2). Structural comparison with *Rm*Bgt17A from *R. miehei* revealed presence of three conserved residues Trp191, Glu192, and Tyr136 in the aglycone subsites proposed to be crucial for the transferase reaction (and not conserved in the homologous *Fb*GH17B, reported as a glucose releasing exo-glucanase), allowing these enzymes to participate in transglycosylation reactions of β-1,3-linked glucans from their respective habitat.

The experimental data demonstrated a distinct specificity of *Ml*GH17B toward β-1,3 linked substrates (DP > 4). The substrate was cleaved two residues from the reducing end of the substrate resulting in Glc_2_ as a leaving group, followed by transfer of the remaining part of the donor substrate to another acceptor molecule of the substrate making a β-1,6-linkage. The resulting products were branched or kinked oligosaccharides, up to sizes exceeding DP20, when using laminaripentaose or laminarihexaose substrates at the activity optimum conditions (pH 6.0 and 20 °C).

## Materials and methods

All materials were purchased from Sigma-Aldrich (Merck) unless otherwise specified. Laminari-oligosaccharides were purchased from Megazyme (Neogen).

### DNA extraction, genome sequencing, and annotation

The bacterial strain ISCAR-4703 was isolated from the coastal, intertidal, marine geothermal Yngingarlindir site located off the coast of the Reykjanes peninsula (Iceland). The strain was cultivated under aerobic conditions at 50 °C on Difco Marine Agar (BD Diagnostics). Genomic DNA was extracted from cells using Epicentre MasterPure Gram Positive DNA Purification Kit (LGC Biosearch Technologies) as described by the manufacturer. The 16S rRNA gene was amplified by PCR with the bacteria-specific primers: F9 (5´-AGTTTGATCCTGGCTCAG-3′; *E. coli* positions 9–27) and R1544 (5´-AGAAAGGAGGTGATCCA-3′; *E. coli* positions 1,544–1,528) with Phusion High-Fidelity DNA Polymerase (New England Biolabs). The amplified 16S rRNA gene was partially sequenced with primer R805 (5´-GACTACCCGGGTATCTAATCC-3′; *E. coli* 805–785) using BrightDye Terminator Cycle Sequencing Kit with ABI 3750 DNA sequencer (PE Applied Biosystems).

For genome sequencing, DNA libraries were prepared using the Nextera XT method (Illumina) and sequenced with the MiSeq System (Illumina). Raw sequences were trimmed for quality using the Trimmomatic v0.39 program (Bolger et al. 2014). A draft genome was assembled using trimmed paired reads and the SPAdes v3.15.2 assembly algorithm (Bankevich et al. 2012). The genome sequences were annotated using the prokaryotic genome annotation server Rapid Annotations using Subsystems Technology (RAST) (Aziz et al. 2008). The number of rRNA and tRNA genes were predicted by the RNAmmer v1.2 tool (Lagesen et al. 2007) and the tRNAscan-SE v2.0 tool (Lowe and Chan 2016) respectively.

### Cloning, protein production and purification

The gene encoding a putative GH17 glycosyl hydrolase annotated in the draft genome of the bacterial strain ISCAR-4703, designated *Ml*GH17B*,* was deposited in the NCBI GenBank with accession number OQ297286. The *MlGH17B* gene was amplified by PCR with Phusion High-Fidelity DNA Polymerase (New England Biolabs).

The forward primer *Ml*GH17B-f2: 5´-**GCCAGCAAGGGCGAG**ATGACCACTAAAGAATTGAGAAG-3′, containing a 5′-linker sequence (bold), and the reverse primer, *Ml*GH17B-bam-h-r: 5´-CGCGGATCCAAATTTTAGTTTTTCATTCTTATCCC-3′ containing a *Bam*HI restriction site (underlined) were used for the amplification. The amplified sequence was cut with *Bam*HI and ligated into a *Sfo*I (blunt end) – *Bam*HI digested pJOE4905 vector (Motejadded and Altenbuchner 2009). Thereby, the 5′ end of the *Ml*GH17B encoding sequence, excluding 58 bp, with the short 5′-linker, was fused in-frame with the vector sequence encoding MBP, and a *S. cerevisiae* ubiquitin-like protein motif (Smt3) for proteolytic cleavage with Ulp1 (Motejadded and Altenbuchner 2009). Furthermore, the 3’end of the sequence was fused with the vector sequence encoding the C-terminal 6 × His-tag. The resulting expression clone, verified by sequencing, was transformed into *Escherichia coli* BL21(DE3).

The expression strain was shake-flask cultivated in LB-Lennox medium at 37 °C inducing heterologous expression at OD_600_ 0.5 with 0.25% (w/v) L-rhamnose for 4 h at 37 °C. The cells were harvested by centrifugation and lysed in lysis buffer (20 mM Tris-HCl pH 7.4, 200 mM NaCl) by ultra-sonication using a UP400S homogenizer (Hielscher Ultrasonics). The lysate was collected after centrifugation at 26,000 × *g* for 20 min at 4 °C, filtered through regenerated cellulose 0.2 μm pore size filters (GE Healthcare Life Sciences), and subjected to an MBP-Trap HP 1 mL (7 × 25 mm) column (GE Healthcare Life Sciences) for affinity purification using ÄKTA start FPLC purification system (GE Healthcare Life Sciences). Bound protein was eluted with maltose gradient up to 10 mM in lysis buffer over 10 column volumes. Fractions containing the recombinant protein were immediately assembled, diluted to final concentration below 1 mg/mL, and stored at 4 °C. The integrity and purity of the protein were analyzed by 4%–15% glycine-SDS-PAGE. The protein concentration was determined considering the absorption coefficient (135,680 M^−1^ cm^−1^) by measuring A_280_ using a NanoDrop 1000 spectrophotometer (Thermo Scientific).

### Melting temperature determination

To determine the thermal unfolding transition, the MBP was cleaved from MBP-Smt3-*Ml*GH17B with Ulp1 and subsequently purified. The pure MBP-Smt3-*Ml*GH17B was incubated with Ulp1 with a fusion-protein/protease ratio of 25:1 (w/w) in the reaction buffer (20 mM Tris–HCl pH 7.4, 10 mM imidazole and 500 mM NaCl) at 30 °C for 1 h. The MBP cleavage reaction after incubation was subjected to an HisTrap HP 1 mL (7 × 25 mm) column (GE Healthcare Life Sciences) collecting purified MBP in a flow through fraction. The integrity and purity of the protein were analyzed by 4%–15% glycine-SDS-PAGE. The protein concentration was determined considering the absorption coefficient (66,350 M^−1^ cm^−1^) by measuring A_280_.

The melting temperature of MBP-Smt3-*Ml*GH17B and MBP at 0.2 mg/mL concentration in lysis buffer with 10 mM maltose were measured applying nanoscale differential scanning fluorimetry (nanoDSF) with Prometheus NT.48 instrument using standard grade capillaries (NanoTemper Technologies), and the results were processed with the PR.ThermControl software (NanoTemper Technologies). The 350/330 nm fluorescence intensity ratio was monitored at 40% excitation power with a temperature gradient 20–95 °C at a ramp rate of 1 °C/min. The inflection point, indicative of a thermodynamic phase change, was considered as melting temperature (T_m_, °C). Furthermore, the onset of the transition (T_onset_, °C) was measured by estimating light scattering, measuring back-reflection light intensity change. The measurements were performed in triplicates.

### Activity optimum determination

The catalytic activity of the enzyme was initially evaluated visualizing reaction products obtained using laminaripentaose (Glc_5_) as the substrate in a broad range of pH 3.5–9 by TLC analysis. Then, a narrower pH range pH 5, 5.5, 6, and 6.5 close to the pH with highest amount of transfer products was selected to confirm the pH optimum. Reaction mixtures of 50 μL were prepared by mixing 5 μL of 20 mg/mL Glc_5_, 5 μL of 500 mM acetate/phosphate buffer, pH 5–6.5, 20 μL of the suitably diluted enzyme in lysis buffer (20 mM Tris-HCl pH 7.4, 200 mM NaCl), and 20 μL of ultrapure water (Milli-Q grade). The reaction mixtures were incubated in the thermal cycler with a temperature gradient 13–49 °C ramping for 2 h, followed by reaction termination at 95 °C for 5 min. Product formation including oligosaccharides with DP2 and DP8 (Glc_2_ and Glc_8_) was analyzed applying HPAEC-PAD. Duplicate samples were analyzed for each condition. The results were plotted, and figures were illustrated using the MODDE v12.1 program (Sartorius).

### MALDI-TOF MS

MALDI-TOF mass spectra were acquired with an autoflex speed MALDI-TOF/TOF (Bruker Daltonics) in positive reflector mode. Samples were diluted with ultrapure water (Milli-Q grade) to receive a total salt concentration less than 10 mM. One microliter of diluted sample was mixed with 0.5 μL of aqueous 10 mg/mL dihydroxybenzoic acid matrix solution. All spectra were externally calibrated using Peptide Calibration Standard II (Bruker Daltonics).

### Purification of transglycosylation products

Ten milliliters of the reaction mixture in 50 mM phosphate buffer, pH 6.0, containing 2 mg/mL of Glc_5_ and 0.4 mg/mL of the enzyme was incubated at 20 °C for 6 h under shaking. The reaction was terminated by incubation at 95 °C for 5 min and the reaction mixture was subsequently filtered through PTFE membrane 0.2 μm pore size filters (Pall). Transglycosylation products were purified applying preparative size-exclusion chromatography (SEC) using Superdex 30 PG size exclusion chromatography media (GE Healthcare Life Sciences) in HiLoad 26/600 column (GE Healthcare Life Sciences). Aliquots of 2 mL of the reaction mixture was loaded and carbohydrates were eluted with ultrapure water (Milli-Q grade) at a flow rate of 0.3 mL/min. Fractions of 0.6 mL were collected, and the carbohydrate content of individual fractions was analyzed by TLC and HPAEC-PAD. Fractions containing a single compound were combined and lyophilized by freeze drying (Labconco).

### NMR spectroscopy

Resolution-enhanced one- and two-dimensional NMR spectra were recorded in D_2_O on an Avance Neo spectrometer (Bruker) equipped with a TCI Prodigy CryoProbe (Bruker) (Utrecht University, The Netherlands) at a temperature of 311 K. Prior to analysis, samples were exchanged twice in D_2_O with an intermediate lyophilization, and then dissolved in 0.5 mL D_2_O. Suppression of the HOD signal was achieved by applying a water-eliminated Fourier transform (WEFT) pulse sequence for one-dimensional NMR experiments. The two-dimensional TOCSY spectra were collected using a composite pulse devised by M. Levitt (MLEV) mixing sequence with 40–150 ms spin-lock times. Multiplicity-edited ^1^H-^13^C HSQC was recorded with 1,536 data points in F2 and 256 in F1. Chemical shifts (δ) are expressed in ppm by reference to internal acetone (δ 2.225 for ^1^H and δ 31.07 for ^13^C).

### Analytical methods

#### Mono- and oligosaccharides quantification

Mono- and oligosaccharides were quantified applying HPAEC-PAD with Dionex ICS-5000 system (Thermo Fisher Scientific). Monosaccharides were detected and quantified using a Dionex CarboPac PA20 (150 × 3 mm, 6 μm) analytical column coupled with a Dionex CarboPac PA20 (30 × 3 mm, 6 μm) guard column (Thermo Fisher Scientific). Monosaccharides were eluted with 0.75 mM NaOH solution at a flow rate of 0.5 mL/min for 30 min, while the column and compartment temperature was maintained at 30 °C (Allahgholi et al. 2020). Monosaccharides were used as standards to detect and quantify analyzed monosaccharides. Oligosaccharides were detected and quantified using a Dionex CarboPac PA200 (250 × 3 mm, 5.5 μm) column coupled with a Dionex CarboPac PA200 (50 × 3 mm, 5.5 μm) guard column (Thermo Fisher Scientific) as described by Allahgholi et al. (Allahgholi et al. 2023). Laminari-oligosaccharides (Glc_2–8_) were used as standards to detect and quantify analyzed oligosaccharides.

#### Thin layer chromatography (TLC)

TLC was performed using TLC Silica gel 60 F_254_ plates (Merck) loading 1 μL of sample and subsequently allowing loaded samples to air-dry. Prepared plates were developed twice with an intermediate drying in a solvent system n-butanol/acetic acid/water, at the solvent ratio 2:1:1 (v/v/v) for Glc_1–8_ oligosaccharides separation or at the solvent ratio 3:2:2 (v/v/v) for Glc_>8_ oligosaccharides separation. Developed plates were air-dried and subsequently treated by spraying with staining solution 5% (v/v) sulfuric acid in methanol supplemented with 1 mg/mL of orcinol. Oligosaccharides were visualized by heating the stained plates at 110 °C on a hotplate.

### Bioinformatics analysis

#### Multiple sequence alignment (MSA)

Sequence similarity searches were made in NCBI BLAST suite (Altschul et al. 1990) with blastp against the non-redundant protein sequence database, UniProtKB/Swiss-Prot database and PDB database using default algorithm parameters. Multiple sequence alignment was performed in Jalview v2.11 program using Clustal Omega v1.2.2 alignment tool (Waterhouse et al. 2009) and graphically presented with the ESpript v3.0 program (Robert and Gouet 2014).

#### Three-dimensional structure modeling

The three-dimensional models for *Ml*GH17B were obtained by running AlphaFold2 using ColabFold v1.5.2: AlphaFold2 (Mirdita et al. 2019; Jumper et al. 2021; Mirdita et al. 2022) (colab.research.google.com/github/sokrypton/ColabFold/blob/main/AlphaFold2.ipynb) which was hosted on Colab notebook.

The model confidence was assessed by the pLDDT score (Jumper et al. 2021) and the top ranked models were used for subsequent analysis. The figure was made using PyMOL v2.5.2 program (Schrödinger).

The three-dimensional structure of *Ml*GH17B was also modeled by homology modeling using the YASARA v21.12.19 program (YASARA Biosciences) as described in Linares-Pastén et al. (Linares-Pastén et al. 2021). The crystal structure of *Rm*Bgt17A (PDB 4WTP, 4WTR, and 4WTS) and *Fb*GH17A (PDB 6FCG) were used as main templates and modeling parameters were set as were applied by Linares-Pastén et al. for modeling the three-dimensional structure of glycosyltransferase enzymes, Glt1 from *P. aeruginosa*, Glt3 from *P. putida*, and Glt20 from *B. diazoefficiens* (Linares-Pastén et al. 2021) (Table S2). The generated hybrid model was subjected to model refinement using default parameters in YASARA program. A simulation cell was designed 2 × 7.5 Å larger than the model along each axis. The simulation cell was filled with water and Na^+^ and Cl^−^ as counter ions. The simulation was run for 500 ps using YAMBER03 force field, and snapshots were stored every 25 ps. The quality of the generated models was assessed through ERRAT, VERIFY3D, and PROCKECK using the UCLA-DOE LAB-SAVES v6.0 server (saves.mbi.ucla.edu). The model was compared with the model generated by AlphaFold2 program.

#### Substrate docking

The top ranked model obtained from AlphaFold2 was subjected to docking experiment with Glc_5_.

Laminaripentaose ligand was built using oligosaccharides building tool available in the YASARA program. The ligand was subjected to MD simulation at physiological pH 7.4 and 298 K for 2 ns using AMBER03 force field in a simulation cell 20 Å larger than the molecule with explicit molecules of water and Na^+^ and Cl^−^ as counter ions. The conformer with the lowest force field energy was selected for docking studies. To perform docking, after the superimposition of the *Ml*GH17B model with the crystal structure of *Rm*Bgt17A (PDB 4WTP) and learning about the location of the active site, AutoDock implemented in YASARA was performed. The quality of the docked model was assessed by checking the conformation of the ligand in the active site, assessing any abnormalities in glycosidic linkages of the ligand, and evaluating fluctuations in RMSD Cα or increasing binding energy during a 50 ns MD simulation.

The three-dimensional model of the enzyme-ligand complex and the active site were graphically presented using the PyMOL program.

## Supplementary Material

Fig_S1_SDS_PAGE_cwae007

Fig_S2_nanoDSF_cwae007

Fig_S3_Product_Profile_Laminarin_cwae007

Figure_S4_modofied_cwae007

Fig_S5_AlphaFold2_YASARA_cwae007

Fig_S6_glucanases_cwae007

Supplementary_materials_cwae007

Quality_assessment_of_Yasara_Model_cwae007

## Data Availability

The data are available on request from the corresponding author.
