# Supplementary figures and images for: Exploring a novel β-1,3-glucanosyltransglycosylase, MlGH17B, from a marine Muricauda lutaonensis strain for modification of laminari-oligosaccharides
Source: Glycobiology. 2024 Jan 25;34(4):cwae007. doi: 10.1093/glycob/cwae007 (PMC11005184; doi:10.1093/glycob/cwae007)

kDa

100

75

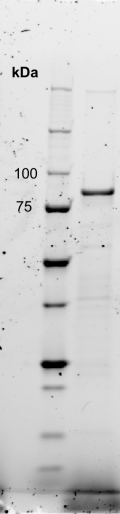

Supplement: Fig_S1_SDS_PAGE_cwae007 [file fig_s1_sds_page_cwae007.pdf]

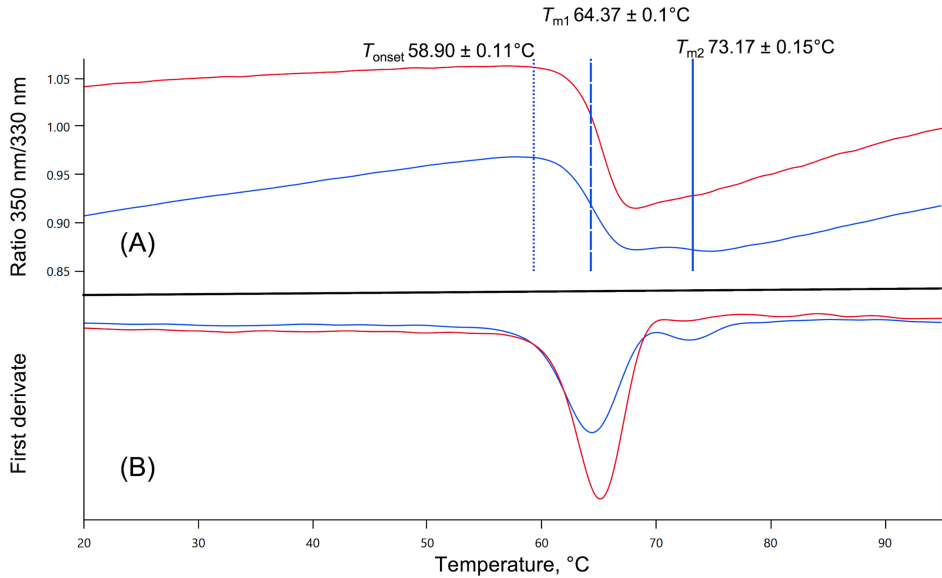

Supplement: Fig_S2_nanoDSF_cwae007 [file fig_s2_nanodsf_cwae007.pdf]

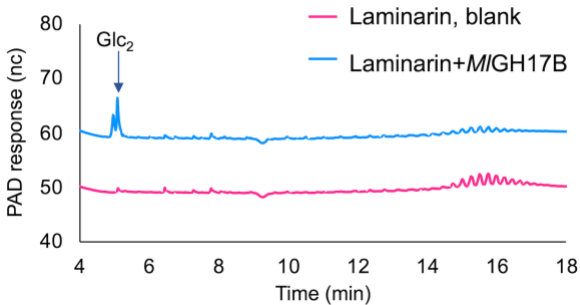

Supplement: Fig_S3_Product_Profile_Laminarin_cwae007 [file fig_s3_product_profile_laminarin_cwae007.pdf]

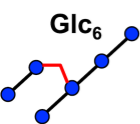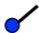

$\beta$ -Glc-(1,3)-

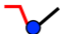

-(1,6)- $\beta$ -Glc-(1,3)-

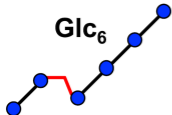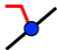

-(1,3-1,6)- $\beta$ -Glc-(1,3)-

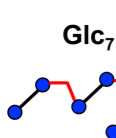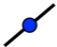

-(1,3)- $\beta$ -Glc-(1,3)-

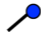

-(1,3)-Glc  $\alpha/\beta$

Supplement: Figure_S4_modofied_cwae007 [file figure_s4_modofied_cwae007.pdf]
