# Supplementary material for: Exploring a novel β-1,3-glucanosyltransglycosylase, MlGH17B, from a marine Muricauda lutaonensis strain for modification of laminari-oligosaccharides
Source: Glycobiology. 2024 Jan 25;34(4):cwae007. doi: 10.1093/glycob/cwae007 (PMC11005184; doi:10.1093/glycob/cwae007)
Supplement: Fig_S5_AlphaFold2_YASARA_cwae007 [file fig_s5_alphafold2_yasara_cwae007.pdf]

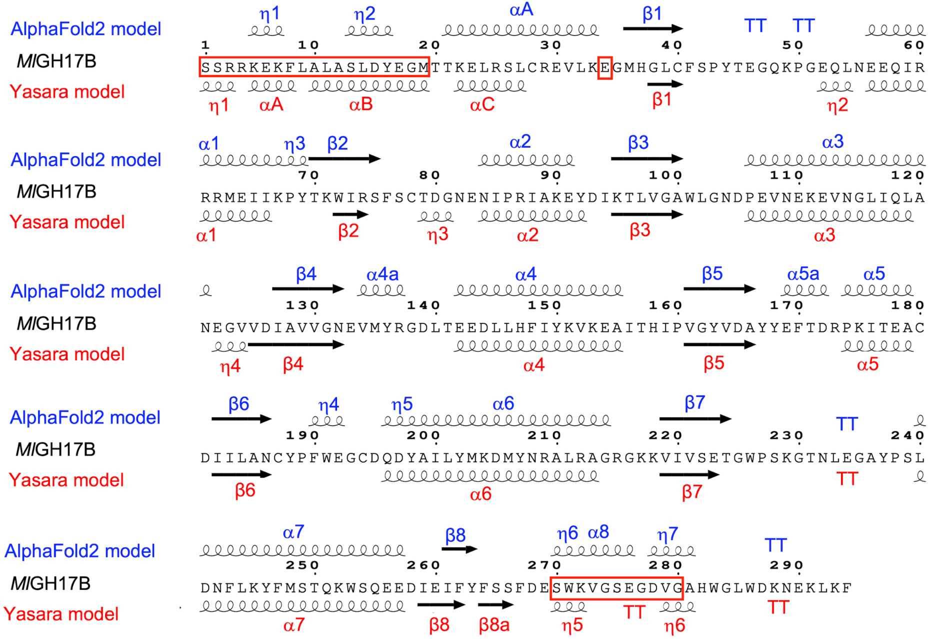
AlphaFold2 model
MlGH17B
Yasara model
η1 η2 αA β1 TT TT
1 10 20 30 40 50 60
SSRRKEKFLALASLDYEGMTTKELRSLCREVLKEGMHGLCFSPYTEGQKPGEQLNEEQIR
η1 αA αB αC β1 η2
α1 η3 β2 α2 β3 α3
70 80 90 100 110 120
RRMEIIKPYTKWIRSFSCTDGNENIPRIAKEYDIKTLVGAWLGNDPEVNEKEVNGLIQLA
α1 β2 η3 α2 β3 α3
β4 α4a α4 β5 α5a α5
130 140 150 160 170 180
NEGVVDIAVVGNEVMYRGDLTEEDLLHFIYKVKEAITHIPVGYVDAYYEFTDRPKITEAC
η4 β4 α4 β5 α5
β6 η4 η5 α6 β7 TT
190 200 210 220 230 240
DIILANCYPFWEGCDQDYAILYMKDMYNRALRAGRGKKVIVSETGWPSKGTNLEGAYPSL
β6 α6 β7 TT
α7 β8 η6 α8 η7 TT
250 260 270 280 290
DNFLKYFMSTQKWSQEEDIEIFYFSSFDESWKVGSEGDVGAHWGLWDKNEKLKF
α7 β8 β8a η5 TT η6 TT
