# Supplementary material for: Exploring a novel β-1,3-glucanosyltransglycosylase, MlGH17B, from a marine Muricauda lutaonensis strain for modification of laminari-oligosaccharides
Source: Glycobiology. 2024 Jan 25;34(4):cwae007. doi: 10.1093/glycob/cwae007 (PMC11005184; doi:10.1093/glycob/cwae007)
Supplement: Fig_S6_glucanases_cwae007 [file fig_s6_glucanases_cwae007.pdf]

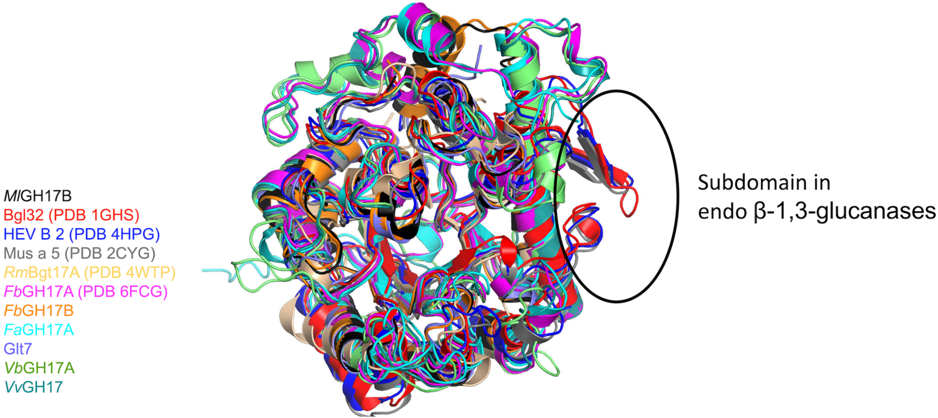

MlGH17B
Bgl32 (PDB 1GHS)
HEV B 2 (PDB 4HPG)
Mus a 5 (PDB 2CYG)
RmBgt17A (PDB 4WTP)
FbGH17A (PDB 6FCG)
FbGH17B
FaGH17A
Glt7
VbGH17A
VvGH17
Subdomain in
endo β-1,3-glucanases
