# Supplementary material for: Exploring a novel β-1,3-glucanosyltransglycosylase, MlGH17B, from a marine Muricauda lutaonensis strain for modification of laminari-oligosaccharides
Source: Glycobiology. 2024 Jan 25;34(4):cwae007. doi: 10.1093/glycob/cwae007 (PMC11005184; doi:10.1093/glycob/cwae007)
Supplement: Supplementary_materials_cwae007 [file supplementary_materials_cwae007.docx]

**Fig. S1.** MBP-Smt3-*Ml*GH17B-6×His purity and integrity determination with SDS-PAGE. Precision Plus Protein Dual Color Standards (Bio-Rad) molecular-mass marker was used to indicate the molecular weight.

**Fig. S2**. Thermostability and thermoaggregation determination of MBP-Smt3-*Ml*GH17B and MBP with nanoscale differential scanning fluorimetry. The melting curve of MBP-Smt3-*Ml*GH17B and MBP proteins are presented in blue and red, respectively. Thermograms were measured at 0.2 mg/mL protein concentration in 20 mM Tris-HCl pH 7.4, 200 mM NaCl, 10 mM maltose. (A) Fluorescence intensity ratio 350 nm/F330 nm. (B) First derivative of fluorescence intensity ratio.

**Fig. S3.** HPAEC–PAD analysis of *Ml*GH17B reaction products obtained with laminarin, at pH 6.0 and 20°C after 24 h of reaction incubation.

**Fig. S4.** Proposed structure for transglycosylation products of *Ml*GH17B acting on laminaritetraose substrate. Glc_6_ and Glc_7_ represent the oligosaccharides with 6 and 7 glucose units. Gα/β represents the reducing end.

**Fig. S5.** Comparison of generated AlphaFold2 and YASARA models of *Ml*GH17B. The AlphaFold2 model (blue) is shown on top of the *Ml*GH17B sequence and the YASARA model (red) is shown under the sequence. The regions with low pLDDT (<90%) in AlphaFold2 model are outlined in red boxes.

**Fig. S6.** Superimposition of *Ml*GH17B to other GH17 enzymes. *Ml*GH17B (black), β-1,3-glucanosyltransglycosylase from *M. lutaonensis* ISCAR-4703; Bgl32 (red), endo-β-1,3-glucanase from *Hordeum vulgare* (PDB 1GHS); HEV B2 (blue), endo-β-1,3-glucanase from *Hevea brasiliensis* (PDB 4HPG); Mus a 5 (gray), endo-β-1,3-glucanase from *Musa acuminata* (PDB 2CYG); *Rm*Bgt17A (light orange), β-1,3-glucanosyltransferase from *Rhizomucor miehei* (PDB 4WTP); *Fb*GH17A (magenta), laminarinase from *Formosa* sp. Hel1_33_131 (PDB 6FCG); *Fb*GH17B (dark orange), laminarinase from *Formosa* sp. Hel1_33_131; *Fa*GH17A (cyan), laminarinase from *Formosa agariphila*; Glt7 (slate blue), glucanosyltransglucosidase from *Azotobacter vinelandii*; *Vb*GH17A (green), laminarinase from *Vibrio breoganii*; *Vv*GH17 (deep teal), endo-β-1,3-glucanase from *Vibrio vulnificus*.

**Table SI.** Contribution of fragments from other models to the hybrid models

| **First residue** | **Last residue** | **Length** | **From model** | **Score** |
| --- | --- | --- | --- | --- |
| 28 | 295 | 268 | 4WTR-A01 | -2.243 |
| 1 | 40 | 40 | 6FCG-D03 | -1.751 |
| 114 | 120 | 7 | 6FCG-D03 | -1.744 |
| 114 | 120 | 7 | 6FCG-D03 | -1.709 |
| 113 | 120 | 8 | 4WTP-A04 | -1.680 |
| 102 | 115 | 14 | 4WTR-A04 | -1.670 |
| 42 | 66 | 25 | 4WTP-A03 | -1.662 |
| 19 | 23 | 5 | 6FCG-D02 | -1.648 |
| 271 | 282 | 12 | 6FCG-D03 | -1.619 |
| 114 | 120 | 7 | 6FCG-D03 | -1.606 |
| 199 | 204 | 6 | 6FCG-D01 | -1.602 |
| 262 | 268 | 7 | 4WTP-A02 | -1.598 |
| 117 | 120 | 4 | 6FCG-D03 | -1.593 |
| 114 | 120 | 7 | 6FCG-D03 | -1.587 |
| 106 | 113 | 8 | 4WTR-A04 | -1.580 |
| 19 | 22 | 4 | 6FCG-D01 | -1.565 |
| 116 | 120 | 5 | 4WTP-A04 | -1.541 |

**Table SII.** Parameters used for the prediction of the three-dimensional structure using YASARA program

| **Parameter** | **Description** |
| --- | --- |
| Modelling speed | Slow |
| PSI-BLAST iterations in template search | 10 |
| Maximum allowed (PSI-) BLAST E-value to consider template | 0.5 |
| Maximum number of templates to be used (Templates Total) | 10 |
| Maximum number of templates with same sequence | 1 |
| Maximum oligomerization state | 4 |
| Maximum number of alignment variations per template | 5 |
| Maximum number of conformations tried per loop | 50 |
| Maximum number of residues added to the termini | 10 |
