# Supplementary material for: Exploring a novel β-1,3-glucanosyltransglycosylase, MlGH17B, from a marine Muricauda lutaonensis strain for modification of laminari-oligosaccharides
Source: Glycobiology. 2024 Jan 25;34(4):cwae007. doi: 10.1093/glycob/cwae007 (PMC11005184; doi:10.1093/glycob/cwae007)
Supplement: Quality_assessment_of_Yasara_Model_cwae007 [file quality_assessment_of_yasara_model_cwae007.docx]

**Quality assessment of YASARA three-dimensional model**

YASARA's calculation of the Z-score -1.46, based on dihedral angles, 1D packing, and 3D packing (Overall = 0.145.Dihedrals + 0.390.Packing1D + 0.465.Packing3D), indicated that the overall quality of the model was satisfactory. The model's quality was assessed through ERRAT, VERIFY3D, and PROCKECK using the UCLA-DOE LAB — SAVES v 6.0 server (saves.mbi.ucla.edu/). The Ramachandran plot generated by PROCHECK [1,2], for the selected model, obtained after model refinement, showed that 93.1% of the amino acids were in the preferred region, 5.4% were in the allowed region, while three residues, Thr79, Thr231, and Glu234 (compromising 1.2% of the residues) were considered as outliers. According to Ramachandran plot, a model that have more than 90% residues located in allowed region is considered as a good quality model, hence in case of *Ml*GH17B over 93% of the residues are located in the allowed region confirming that the model has a satisfactory quality [3,4]. In addition, overall quality factor of the model calculated 98.25 % by ERRAT indicating a good quality of the model as, according to this tool, good high-resolution structures generally produce values around 95% or higher. For lower resolutions (2.5 to 3A) the average overall quality factor is around 91% [5].

1. Laskowski, R. A., Rullmannn, J. A., MacArthur, M. W., Kaptein, R., and Thornton, J. M. (1996) AQUA and PROCHECK-NMR: programs for checking the quality of protein structures solved by NMR. *J Biomol NMR* **8**, 477-486

2. Laskowski, R. A., MacArthur, M. W., Moss, D. S., and Thornton, J. M. (1993) PROCHECK: a program to check the stereochemical quality of protein structures. *J. Appl. Crystallogr.* **26**, 283-291

3. Laskowski, R. A., Jabłońska, J., Pravda, L., Vařeková, R. S., and Thornton, J. M. (2018) PDBsum: Structural summaries of PDB entries. *Protein Sci* **27**, 129-134

4. Laskowski, R. A., Hutchinson, E. G., Michie, A. D., Wallace, A. C., Jones, M. L., and Thornton, J. M. (1997) PDBsum: a web-based database of summaries and analyses of all PDB structures. *Trends in Biochemical Sciences* **22**, 488-490

5. Colovos, C., and Yeates, T. O. (1993) Verification of protein structures: patterns of nonbonded atomic interactions. *Protein Sci* **2**, 1511-1519
